# Identification, characterization of Apyrase (*APY*) gene family in rice (*Oryza sativa*) and analysis of the expression pattern under various stress conditions

**DOI:** 10.1371/journal.pone.0273592

**Published:** 2023-05-10

**Authors:** Aniqua Tasnim Chowdhury, Md. Nazmul Hasan, Fahmid H. Bhuiyan, Md. Qamrul Islam, Md. Rakib Wazed Nayon, Md. Mashiur Rahaman, Hammadul Hoque, Nurnabi Azad Jewel, Md. Ashrafuzzaman, Shamsul H. Prodhan

**Affiliations:** 1 Department of Genetic Engineering and Biotechnology, School of Life Sciences, Shahjalal University of Science and Technology, Sylhet, Bangladesh; 2 Plant Biotechnology Division, National Institute of Biotechnology, Ganakbari, Ashulia, Savar, Dhaka, Bangladesh; 3 Institute of Epidemiology, Disease Control and Research (IEDCR), Dhaka, Bangladesh; Texas Tech University, UNITED STATES

## Abstract

Apyrase (*APY*) is a nucleoside triphosphate (NTP) diphosphohydrolase (NTPDase) which is a member of the superfamily of guanosine diphosphatase 1 (GDA1)—cluster of differentiation 39 (CD39) nucleoside phosphatase. Under various circumstances like stress, cell growth, the extracellular adenosine triphosphate (eATP) level increases, causing a detrimental influence on cells such as cell growth retardation, ROS production, NO burst, and apoptosis. Apyrase hydrolyses eATP accumulated in the extracellular membrane during stress, wounds, into adenosine diphosphate (ADP) and adenosine monophosphate (AMP) and regulates the stress-responsive pathway in plants. This study was designed for the identification, characterization, and for analysis of *APY* gene expression in *Oryza sativa*. This investigation discovered nine *APY*s in rice, including both endo- and ecto-apyrase. According to duplication event analysis, in the evolution of *OsAPY*s, a significant role is performed by segmental duplication. Their role in stress control, hormonal responsiveness, and the development of cells is supported by the corresponding cis-elements present in their promoter regions. According to expression profiling by RNA-seq data, the genes were expressed in various tissues. Upon exposure to a variety of biotic as well as abiotic stimuli, including anoxia, drought, submergence, alkali, heat, dehydration, salt, and cold, they showed a differential expression pattern. The expression analysis from the RT-qPCR data also showed expression under various abiotic stress conditions, comprising cold, salinity, cadmium, drought, submergence, and especially heat stress. This finding will pave the way for future *in-vivo* analysis, unveil the molecular mechanisms of *APY* genes in stress response, and contribute to the development of stress-tolerant rice varieties.

## Introduction

Apyrase (*APY*), which is a class of nucleoside triphosphate (NTP) diphosphohydrolase (NTPDase) and a member of the superfamily of GDA1-CD39 (guanosine diphosphatase 1- cluster of differentiation 39) nucleoside phosphatase [1], mediates the concentrations of NTP (nucleoside triphosphate) and NDP (nucleoside diphosphate) [2], especially of ATP (adenosine triphosphate) and ADP (adenosine diphosphate) and catalyzes their breakdown and converts them into ADP and AMP (adenosine monophosphate) [3, 4]. ATP, the general source of energy in every cell, functions in a stress-dependent fashion since it promotes cell growth and development when present in lower concentrations. When the plant cells encounter wound [5], pathogen elicitors ([6, 7], cell growth [8], abiotic stress ([9, 10], touch [9, 11], they discharge ATP into the extracellular matrix (ECM) where it becomes known as eATP (extracellular ATP). These stimuli typically occur simultaneously and involve hormonal and signaling pathway cross-talk [12, 13]. Survival of plants [8], development [4, 6, 14] gravitropism [15], plant defense strategy [16], cellular apoptosis [17], stress responsiveness [3, 5, 9, 18], are controlled by eATP. When this happens, the level of eATP rises, which has a number of detrimental effects on cells, such as slowing cell proliferation [19], ROS (reactive oxygen species) production, NO (nitric oxide) burst, as well as triggering apoptosis [20]. At this point, *APY* comes into action by breaking down eATP, which helps plants in cell survival, growth, development, and face off several stresses [21].

The presence of five apyrase conserved regions (ACRs) is a defining trait of NTPDase family proteins [22], and APYs can be separated as ecto-and endo-APY, as per the subcellular localization [23]. Ecto-APYs are situated on the cell’s surface, and the ones located on cytoplasmic organelles are generally called endo-apyrases. Many apyrase genes include transmembrane domains at their N- and C- terminals [24] and generally feature amino acid glycosylation, which is necessary to ensure proper folding of proteins, targeting at the membrane, cellular distribution, and enzymatic function [1]. However, APYs are not similar to ATPases such as APYs can employ several divalent cofactors like Mn^2+^, Zn^2+^, Ca^2+^, and Mg^2+^, in contrast to ATPases, which employ Mg^2+^ as a cofactor [25]. It also demonstrates insensitivity towards alkaline phosphatase as well as to certain types of ATPase inhibitors [26].

There are several plant species in which *APY*s were discovered, including potato [27, 28], *Arabidopsis* [2], soybean [29], cotton [30], wheat [23], peanut [31]. Especially in *Arabidopsis*, wheat and peanut, the gene family has been intensively studied. Seven *APY* members in *Arabidopsis* have been identified and described [2]. AtAPY1 and AtAPY2 are endo-APYs as they are found in the Golgi body. They control various cellular phases [2, 32], and their mutation can dramatically increase extracellular ATP (eATP), which confirms that APYs located intracellularly might also be able to control eATP homeostasis [4, 19]. Others are ecto-APYs, and there have been many pieces of evidence about the involvement of ecto-APYs in the hydrolyzing of eATP [17]. Two other AtAPYs, AtAPY6 and AtAPY7, are also essential to pollen production by controlling the synthesis of polysaccharides [33]. Nine *APY* members are identified in wheat with different expression patterns when subjected to various stresses and even in different tissues [23]. In many species, *APY*s have been found to have involvement in different stress tolerance, such as tolerance towards salinity and drought conditions in *Arabidopsis* after overexpression of *PeAPY1* and *PeAPY2* [34], tolerance to waterlogging in soybean [35]. *PsAPY*, when expressed ectopically, has been reported to exhibit resistance in tobacco to pathogen attack [36]. However, the total signaling pathway working behind the activity of *APY* is still not explained.

Rice is among the world’s most widely grown as well as consumed cereals. The majority of the world relies on rice as a main meal [37]. Agricultural production is at risk due to climate change. Likewise, rice production is being hampered due to climate anomalies, including submergence, less rainfall, cold weather, increasing salinity levels, and many more. Fungal and bacterial blight are two of the most severe and prevalent rice diseases that reduce annual rice production [38]. Developing stress-resistant rice may increase yield tremendously as genome-editing and genetic transfer technologies are improving rapidly; these methods have made it possible to grow crops that can withstand stress. A thorough genome-wide investigation on stress-associated gene families using bioinformatics approaches has been accomplished thanks to the recent availability of information on the genomes of various crop species. Rice is the first food grain of which the entire genome sequence is available [38]. It provides a chance to locate the genes and systematically categorize the genes and biochemical pathways essential for expanding rice production, conferring tolerance towards several stresses, as well as enhancing the product’s quality.

Different families of genes such as *PYL* [39], *NAC* [40], *GRAS* [41], *MYB* [42], *WRKY* [43], *DREB* [44] are reported in rice as gene families associated with responsiveness towards stress. When exposed to various stress environments, many genes have been observed to exhibit particular responses. For example, cold, high salinity, and drought are associated with the induction of *OsNAC6/SNAC2* [45]. During dehydration, the HKT-1 protein is required for cell osmoregulation and the maintenance of turgor [46]; the HSP20 family provides tolerance to prolonged heat stress and resistance to different environmental stresses [45]. The CRT/DREBP protein family is mainly regulated by cold stress; on the other hand, DREBP (DRE-binding protein) family induction is caused by drought and high salt stress [47]. Overexpression of different genes responsive to stress in rice could increase the tolerance level when subjected to such stress. Such genes include *OsNHX1* in salt stress [48], *OsDREB* under drought conditions [49], *Sub1A* when subjected to submergence stress [50]. Identifying and characterizing such genes responsive to stresses and applying molecular methods could pave the way toward developing rice varieties tolerant of several stresses.

Since there is ample evidence about the role of *APY*s during the stress response, their identification and characterizing in plants might help find new development sites for strengthening tolerance towards several stresses through different gene modification methods. There is an absence of studies about *APY*s in rice. The purpose of this research is to characterize *APY* genes in *O*. *sativa* and provide insight into their function throughout development and in response to stress by doing a genome-wide identification and analyzing their expression profiles. Rice *APY* family members are identified using bioinformatics techniques throughout this study. We have further studied the functional characterization, including phylogenetic relationship analysis with the *APY*s from *Arabidopsis*, peanut and wheat. Both RT-qPCR and RNA-seq data are utilized in profiling the pattern of gene expression of the *OsAPY*s and suggest their importance in the process of development as well as response to stress. Conclusively, this result will provide a foothold in further analyzing the roles of *OsAPY* in different stress conditions and in identifying targets to enhance rice’s tolerance level under various stresses.

## Materials and methods

### Identification of *APY* family members in *Oryza sativa*

The TAIR database (https://www.arabidopsis.org/) [51] was employed to get the sequences of the proteins for the seven genes (At3g04080, At5g18280, At1g14240, At1g14230, At1g14250, At2g02970, and At4g19180) of *Arabidopsis thalian*a that belong to the apyrase gene family [2]. On Phytozome v12.1 (https://phytozome.jgi.doe.ov) [52], these sequences were used to run a BLASTp search against the *Oryza sativa* v7 jGI (Rice) dataset. They were also used for the BLASTp search against the NCBI protein database (https://www.ncbi.nlm.nih.gov/protein) and the Rice genome annotation project (http://rice.plantbiology.msu.edu/) [53] separately so that no putative member was missed out. The best hits from these databases were subjected to domain analysis via InterPro (http://www.ebi.ac.uk/interpro/) [54] to ensure the presence of the GDA1-CD39 domain, which is a characteristic feature of APYs. Entries having this domain were chosen for multiple sequence alignment, which was conducted via MEGA X (Molecular Evolutionary Genetics Analysis) software [55]. Their multiple sequence alignment was visualized via GeneDoc version 2.7 [56] to search for the presence of 5 ACRs [22]. Sequences that contained these 5 ACRs were chosen as members of the apyrase family. To confirm that all members of the apyrase family were included, those sequences were submitted as input sequences for a BLAST analysis in Phytozome [52]. The coding sequence (CDS), genomic, and peptide sequence of the *OsAPY*s were obtained from Phytozome [52].

### Analysis of the physicochemical properties

For assessment of the physicochemical characteristics of the genes, the theoretical isoelectric point (pI), index of instability, grand average of hydropathy (GRAVY), as well as molecular weight of the proteins were determined. It was done via ProtParam hosted by ExPASy (https://web.expasy.org/protparam/) [57] by uploading the protein sequence. By uploading the protein sequences to WoLF PSORT (https://wolfpsort.hgc.jp/) [58] and CELLO v.2.5 (http://cello.life.nctu.edu.tw/) [59], the subcellular distribution of the proteins was determined.

### Analysis of conserved motifs and gene structure

MEME version 5.3.0 (Multiple Em for Motif Elicitation; http://meme-suite.org) [60] was employed to investigate the conserved motifs on proteins in classic mode, and the motif number was determined to be 10. The minimum width was kept to 6, and the maximum one was 50; each motif’s highest and lowest sites were set to 600 and 2 accordingly. Depending on the appearance frequency of the motifs in MEME [60], they were numbered sequentially and positioned beside their corresponding OsAPYs according to their phylogenetic groups. In order to determine their role, each of the ten detected motifs was evaluated via Pfam (http://pfam.xfam.org/) [61].

*OsAPY* CDS sequences were compared against their respective genomic sequences lacking the UTR (untranslated region) to assess gene structure. Gene structure was determined using the GSDS 2.0 (Gene Structure Display Server) tool (http://gsds.gao-lab.org/) [62], and this web server graphically depicted the gene structure of *OsAPY* genes.

### Analysis of phylogenetic relationship

TAIR [50] along with EnsemblPlants (https://plants.ensembl.org) [63] database and Peanut Genome Recourse (PGR) database (http://peanutgr.fafu.edu.cn) [64] was employed to get the peptide sequences of *APY*s from *Arabidopsis thaliana*, *Triticum aestivum* and *Arachis hypogaea* correspondingly. An unrooted phylogenetic tree was created utilizing MEGA X [55]. It was generated using protein sequences from *Oryza sativa*, *Arabidopsis thaliana*, *Triticum aestivum* and *Arachis hypogaea* [65] to keep the rates uniform across sites. It was visualized using the web tool iTOL (https://itol.embl.de/) [66].

### Prediction of gene duplication

PGDD (Plant genome duplication database) (http://chibba.agtec.uga.edu/duplication/) [67] was employed to examine the gene duplication events and to find out the orthologous and paralogous gene pairs. PGDD [67] provided the Ka (synonymous substitution rate) and Ks (non-synonymous substitution rate) values for the duplicated gene pairs. Using the Ks values and a clockwise rate of synonymous substitution (λ) of 1.5 x 10^−8^, the approximate timing of duplication was computed following the formula T = Ks/2λ [68]. A circle plot of the paralogous genes was created using TBTools (https://github.com/CJ-Chen/TBtools/releases) [69].

### Study of the cis-regulatory elements

The sequence located 1000 bp upstream of *OsAPY*s was retrieved from the genomic sequence of rice using Phytozome [52] for investigating the cis-elements and their function. PlantCARE (http://bioinformatics.psb.ugent.be/webtools/plantcare/htmL/) [70] was utilized for predicting the cis-elements. Cis elements were categorized into different groups, and their functions were identified from the literature review. TBTools [69] was utilized to create a visual description of the cis-regulatory elements.

### Analysis of chromosomal distribution

Information from rice genome sequences was used to build a chromosomal organization of *OsAPY* genes based on their location. Phytozome [52] provided details on the number of chromosomes, gene loci, as well as length for the physical map. MapChart (http://www.joinmap.nl) [71] software was used to create a rudimentary physical map of the *OsAPY* gene family, illustrating its location and distribution.

### Specification of *OsAPY* targeting miRNAs

For the identification of miRNAs which target *APY* genes in rice, psRNATarget (http://plantgrn.noble.org/psRNATarget/) [72] was used, and it was tested against every mature rice miRNAs present in the database of miRbase [73]. Using Cytoscape (https://cytoscape.org/) [74], the predicted miRNAs were deployed to build a network. To perform the analysis of pre-computed expression of the detected miRNAs, miRid was employed to search against the rice datasets of the PmiRExAt (http://pmirexat.nabi.res.in/) [75] and a heatmap was generated via GraphPad Prism 9.0.0 (https://www.graphpad.com/company/) [76].

### Identification of SNPs in *APY* genes

The sequences of *APY* genes from 11 rice varieties with different genotypes based on responses towards abiotic stress [39, 77] were compared to the reference Nipponbare sequence using the Rice SNP-Seek database (https://snp-seek.irri.org/index.zul) [78]. SNPs that could be predominantly attributed to variations in the peptide sequence were recognized throughout the genotypes and were termed single amino acid polymorphisms (SAPs). Employing the Rice Stress-Resistant SNP Database, the detected SNPs were studied to determine which ones contributed to the development of specific stress resistance in the rice varieties [79].

### Analysis of secondary and tertiary structure of proteins

The secondary structures were generated utilizing the STRIDE program (http://webclu.bio.wzw.tum.de/stride/) [80]. The number and percentage of α helices, extended beta-sheet, turns, coils, and 310-helix were displayed in various colors as part of the structural study. The membrane-spanning motif was investigated by using default parameters of the online tool TMHMM server v.2.0 (http://www.cbs.dtu.dk/services/TMHMM/) [81].

The tertiary structure was generated using the RoseTTAFold method of the Robetta server (https://robetta.bakerlab.org/) [82]. Refinement of the created structures was done via the GalaxyRefine web server (http://galaxy.seoklab.org/refine) [83], and then the energy minimization was done using Swiss-PdbViewer v4.1 (http://www.expasy.org/spdbv/) [84]. PROCHECK (https://servicesn.mbi.ucla.edu/PROCHECK/) [85] and ERRAT (https://servicesn.mbi.ucla.edu/ERRAT/) [86] servers were further used for the validation of the structure. For the assessment of Z-scores and energy plots, ProSA-web (https://prosa.services.came.sbg.ac.at/prosa.php) [87] was utilized. Discovery Studio software was finally used to visualize the tertiary protein structures [88].

### Analysis of protein-ligand docking

Molecular docking is an *in-silico* strategy for assessing the binding affinity of a ligand to a receptor molecule. Since ATP is a vital ligand of APY proteins, it was chosen as the ligand for the study. HDOCK server (http://hdock.phys.hust.edu.cn/) [89] was used as the docking tool to conduct the docking analysis between the OsAPY proteins and ATP. It was done using the default parameters. The 3D structure of ATP was downloaded from the PubChem database (PubChem CID: 5957). After docking, the top 10 solution complex generated via HDOCK [89] were downloaded, and their structures were further analyzed and validated using PDBsum (http://www.biochem.ucl.ac.uk/bsm/pdbsum) [90], PROCHECK [85] and ERRAT [86] servers. The best structure was selected, and it was aligned and visualized. Then finally, the protein and ligand residue interaction was studied using Discovery Studio [88].

### Study of expression profiling of *OsAPY*s in rice using RNA-seq data

Rice Genome Annotation Project [54] was utilized to collect the data for the expression of *APY* genes under several tissues. Accordingly, the RNA-seq expression values were listed. GraphPad Prism 9.0.0 [76] software was used to create a heat map from the data. GENEVESTIGATOR (https://genevestigator.com/gv/) [91] provided the pattern of *OsAPY* expression in various stresses, and the log2 values of relative expression were used to generate the heat map via GraphPad Prism 9.0.0 [76].

### Plant germination, treatment, and total RNA isolation

The healthy, high-quality and mature seeds of BRRI dhan28 were obtained from the Bangladesh Rice Research Institute (BRRI) and used in this experiment. They were properly cleaned before being placed in a petri dish with tissue paper that had been soaked in water. The seedlings that had sprouted were relocated to the hydroponic culture system after three to four days. In the culture room, a controlled environment was managed, including a temperature of 25±2°C with a photoperiod consisting of 16 hours of light followed by 8 hours of darkness and 1500–2000 lux light intensity. After 20 days, the seedlings were subjected to different stresses for 18–20 hours which included cadmium stress (100mM CdCl_2_ dissolved in distilled water), cold (4°C), salinity (100mM NaCl dissolved in distilled water), submergence, drought (3 mg/L polyethylene glycol dissolved in distilled water), heat stress (42°C). As control, seedlings that had been untreated were utilized. After the treatment, fresh young leaves were collected and washed several times with 70% alcohol and distilled water in order to extract the RNA. Using Invitrogen^TM^ TriZOL^TM^ reagent (Thermo Fisher Scientific Corporation, USA), total RNA was isolated from leaves. The extracted RNA was subsequently processed with DNase I of Invitrogen^TM^ DNA-free^TM^ DNA Removal Kit (Thermo Fisher Scientific Corporation, USA) to remove the genomic DNA contamination. Finally, the GoScript^TM^ Reverse Transcription System (Promega Corporation, USA) was utilized to synthesize the complementary DNA (cDNA). These procedures were carried out per the protocol of the manufacturers.

### Analysis of gene expression under abiotic stress conditions using RT-qPCR data

Primer3 v.0.4.0 (https://bioinfo.ut.ee/primer3-0.4.0/) [92] was used to generate primers for the RT-qPCR, keeping the product length between 208 and 220 bp. The real-time PCR was done using GoTaq® qPCR Master Mix (2X) (Promega Corporation, USA) on CFX96^TM^ Real-Time PCR Detection System (BioRad). For the reference gene, eukaryotic elongation factor 1 alpha (*eEF-1α*) was chosen [93]. Each set of gene-specific primers took up 1 μL of the 15 μL reaction mixture. It also comprised 7.5 μL of GoTaq® qPCR Master Mix (2X), 2 μL of cDNA (10 times diluted), and 3.5 μL of nuclease-free water. In order to carry out each of the reactions, the following conditions were applied: at 95°C initial denaturation for 10 min preceding 40 cycles of denaturation at 95°C for 15s, annealing for 30s, and extension at 72° C for 40s. The annealing temperature was 55.4°C for *OsAPY2* and *OsAPY4*; 56.4°C for *OsAPY1* and *OsAPY5*; 57.4°C for *OsAPY3*, *OsAPY6*, and *OsAPY8*; 58.4°C for *OsAPY7* and *OsAPY9*; 55.7°C for *eEF-1α*. Each sample was subjected to three separate experiments and melting curve analysis was performed following PCR amplification. The delta-delta Ct value approach [94] was utilized in calculating the ratio of relative expression. Technical replication was used to find the mean value of expression at different treatments. MS Office 365 and GraphPad Prism 9.0.0 [76] were used to analyze the data. A one-way ANOVA, followed by a Bonferroni post hoc test, was used to assess the significant differences (P ≤ 0.05). To represent the significant differences, different means were labeled with different number of stars (*).

## Results

### Identification of *APY* family members in rice and study of their physicochemical properties

Rice was found to contain nine members of the *APY* gene family. To BLAST against the rice genome, the *Arabidopsis APY*s were utilized as reference sequences. Upon performing BLASTp searches against the *Oryza sativa*, a total of 19 hits were found containing the GDA1-CD39 domain. All the domain annotations were confirmed by sequence analysis in InterPro [54].

These 19 transcripts belonged to a total of 12 genes. The primary transcripts annotated by Phytozome [52] were selected as the representative transcripts of the genes with alternative splice forms. Nine of the 12 genes were found to possess all five Apyrase Conserved Regions (ACRs) (S1 Fig); hence they were designated Apyrase genes. For the naming of the genes (*OsAPY1*-*OsAPY9*), the prefix ’Os’ for *Oryza sativa* was used, followed by ’APY’ for Apyrase, and then the sequential number that corresponded to their chromosome number and location was used. Table 1 lists the physicochemical properties of all representatives of the *OsAPY* Family.

**Table 1:** Physicochemical properties of the members of the apyrase gene family in rice (*O*. *sativa*).

Gene Name	Locus Name	Length			MW (Da)	pI	Instability Index	GRAVY Value	Localization
		Genomic (bp)	CDS (bp)	Protein (aa)					
*OsAPY1*	LOC_Os03g21120	5548	1470	489	52799.01	5.72	39.76 (Stable)	-0.09	Cp^a^^b^
*OsAPY2*	LOC_Os03g26080	3847	1527	508	55346.06	9.1	53.33 (Unstable)	-0.204	PM^a^^b^
*OsAPY3*	LOC_Os07g48430	4260	1404	467	50437.74	8.97	36.76 (Stable)	-0.136	Mt^a^, Cp^b^
*OsAPY4*	LOC_Os08g33850	4217	1629	542	59596.12	9.34	48.76 (Unstable)	-0.149	PM^a^, ER^b^
*OsAPY5*	LOC_Os10g21000	4624	2109	702	77242.54	9.18	43.81 (Unstable)	-0.171	PM^a^^b^
*OsAPY6*	LOC_Os11g03270	6059	1647	548	59542.4	7.96	36.68 (Stable)	-0.077	Cp^a^^b^
*OsAPY7*	LOC_Os11g03290	3902	1374	457	49622.22	5.44	31.59 (Stable)	-0.179	Cp^a^^b^
*OsAPY8*	LOC_Os11g25260	7876	1428	475	50321.86	5.83	37.31 (Stable)	-0.12	Cp^a^^b^
*OsAPY9*	LOC_Os12g02980	3138	1356	451	48900.8	8.01	30.98 (Stable)	-0.155	Cp^a^, ER^b^

CDS- Coding sequence, MW- Molecular weight, pI- Isoelectric point, GRAVY- Grand average of hydropathy, Cp- Chloroplast. PM- Plasma membrane, Mt- Mitochondria, ER- Endoplasmic reticulum.

^a^Subcellular localization according to CELLO,

^b^subcellular localization according to WoLF PSORT.

The theoretical pI of proteins belonging to the apyrase gene family ranged between 5.44 and 9.34, with an average of 7.73. *OsAPY*4 had the highest and *OsAPY*7 had the lowest pI value. The proteins had molecular weights ranging from 48900.8 Da to 77242.54 Da belonging to *OsAPY*9 and *OsAPY*5, respectively. The average molecular weight of the proteins was 55978.8 Da. The GRAVY value for all the proteins was negative, and as per the data, all protein molecules were hydrophilic [95]. Six genes had an instability index lower than 40, meaning they were stable. It revealed that 67% of the genes were stable, and 33% of them were unstable.

For the prediction of subcellular location, two different tools were used, and per the subcellular localization, both ecto- and endo-APYs were present in this gene family [23]. Their results varied in OsAPY3, OsAPY4, and OsAPY9. According to CELLO [59], they were located on mitochondria, plasma membrane, and chloroplast. On the other hand, according to WoLF PSORT [58], their subcellular localization was on chloroplast for OsAPY3 and endoplasmic reticulum for OsAPY4 and OsAPY9. The predicted subcellular localization of the proteins according to two different tools is given in Table 1.

### Analysis of conserved motifs and gene structure

**Fig 1:**
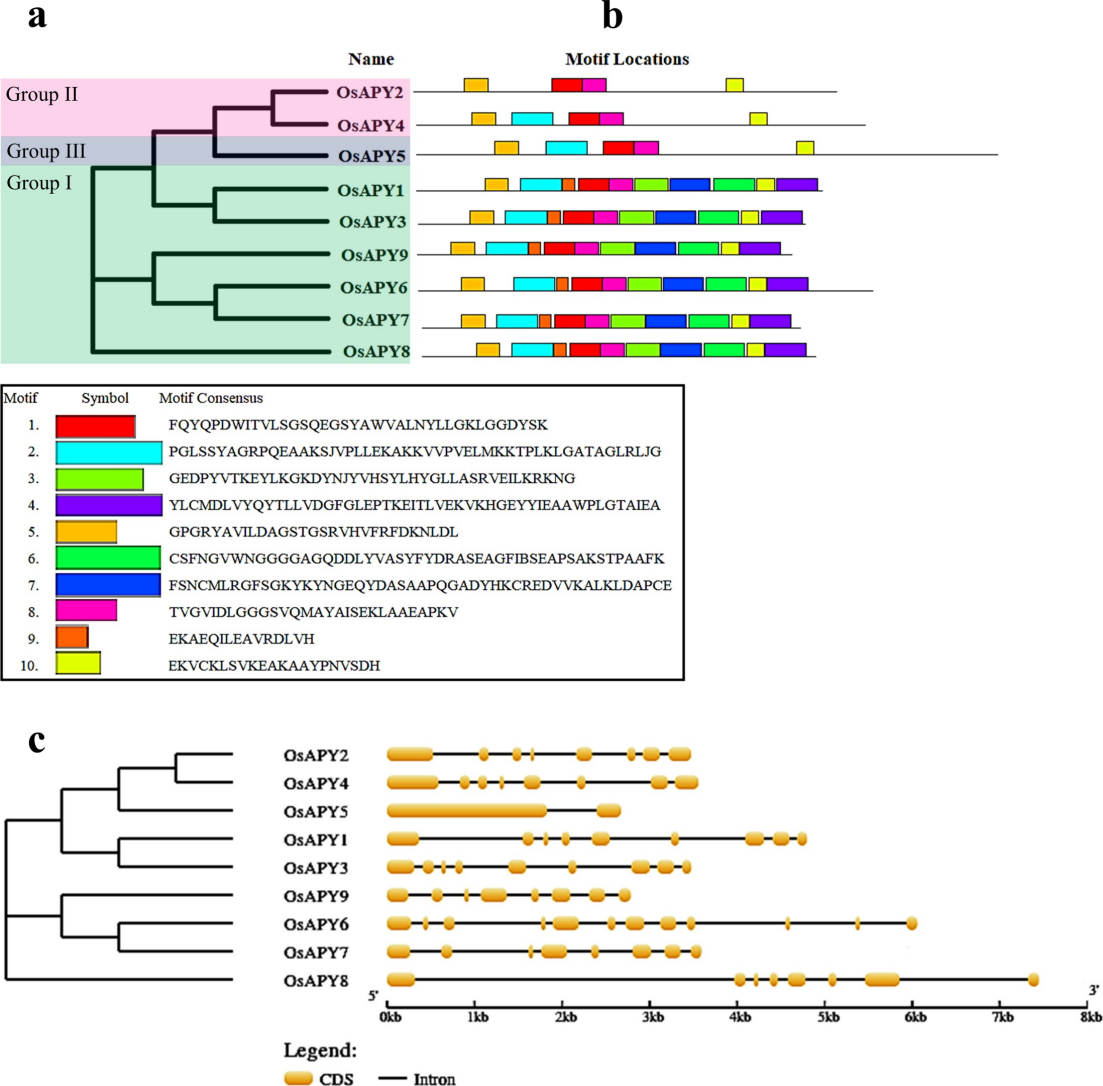
Schematic depiction of phylogenetic relationship, gene structure, and conserved motifs of *APY* genes. a- Phylogenetic relationship among *OsAPY*s generated via maximum likelihood approach. The green, pink and blue boxes represent groups I, II, and III, respectively. b- OsAPY conserved motif distribution. Ten varying-colored boxes represent the motifs. The legend below shows the corresponding motif’s protein sequence. c- Gene structure of *APY*s. Yellow color boxes specify exons, and introns are depicted by lines. The exon-intron lengths can be calculated following the scale shown below.

Ten conserved motifs in total were discovered, which were numbered 1–10 (Fig 1B). 4 motifs among them (motif 1, 5, 8, and 10) were conserved and existed in all the OsAPYs. Members of each phylogenetic group had similar motif distribution, which affirms the classification of groups. All the members of group I in the phylogenetic tree (Fig 1A) had the same motif distribution, containing all the ten motifs. On the other hand, the members of group II in the phylogenetic tree, OsAPY2, and OsAPY4, contained almost similar numbers of motifs except motif 2, which was present in OsAPY4 only. Though only OsAPY5 belonged to group III, its motif distribution was the same as OsAPY4 despite being in different phylogenetic groups. Functions of these motifs were also analyzed, but there was no information on the function of motif 9 and 10. Their analysis revealed that the remaining eight motifs belonged to the GDA1/CD39 (nucleoside phosphatase) family (S1 Table).

The exon and intron arrangement of *OsAPY*s was studied in order to better comprehend their structure. The total number of coding sequences (CDS) or exons and introns of the *OsAPY* genes were 2–12 and 1–11, respectively (Fig 1C), and there was no intronless gene. *OsAPY5* contained the lowest number of exons and introns, which were two exons and one intron, and on the other hand, *OsAPY6* contained the highest number of exons and introns, which was 12 exons and 11 introns.

### Analysis of phylogenetic relationship in *OsAPY* family

**Fig 2:**
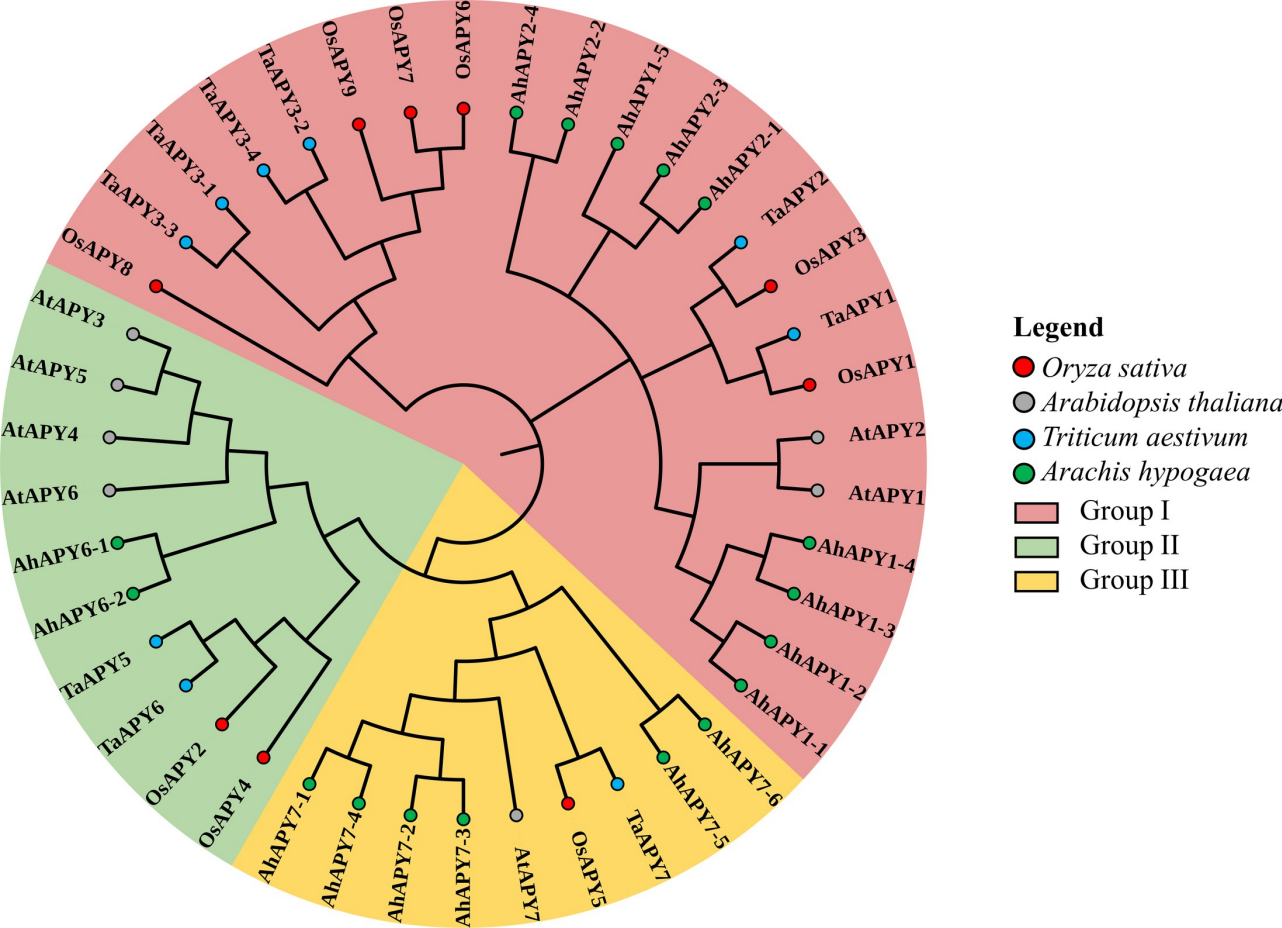
Phylogenetic analysis of the *APY*s in rice, *Arabidopsis*, peanut and wheat. The MEGA X software was employed to generate a phylogenetic tree by utilizing the maximum likelihood approach along with 1000 bootstrap replications. Genes were divided into three different groups, each marked with a different color.

The maximum likelihood method was utilized to create an unrooted phylogenetic tree to study the evolutionary relationship between the *APY* genes. It was done using the peptide sequences of seven apyrase homologs from *Arabidopsis thaliana* (*AtAPY*) [2], nine apyrase homologs from *Triticum aestivum* (*TaAPY*) [23], seventeen homologs from *Arachis hypogaea* (*AhAPY*) [31] and the nine identified apyrase homologs from *Oryza sativa* (*OsAPY*). This phylogenetic tree (Fig 2) demonstrated that the nine *OsAPY* genes could be split into three distinct groups referred to as I, II, and III. There were already three distinct groups for the *AtAPY*, *TaAPY* and *AhAPY* genes [23]. No *OsAPY* gene was classified into the new group. Among the 9 *OsAPY* genes, *OsAPY1*, *OsAPY3*, *OsAPY6*-*OsAPY9* belonged to group I. *TaAPY1*-*TaAPY3*.*4*, *AhAPY1-1-AhAPY1-5*, *AhAPY2-1-AhAPY2-4* and *AtAPY1*, *AtAPY2* also were in group I. *OsAPY2*, and *OsAPY4* fell in group II. Group II also contained *TaAPY5*, *TaAPY6*, *AtAPY3*-*AtAPY6*, *AhAPY6-1-AhAPY6-2*. Group III consisted of *OsAPY5*, *AtAPY7*, and *TaAPY7*, *AhAPY7-1-AhAPY7-6*.

### Analysis of gene duplication

**Fig 3:**
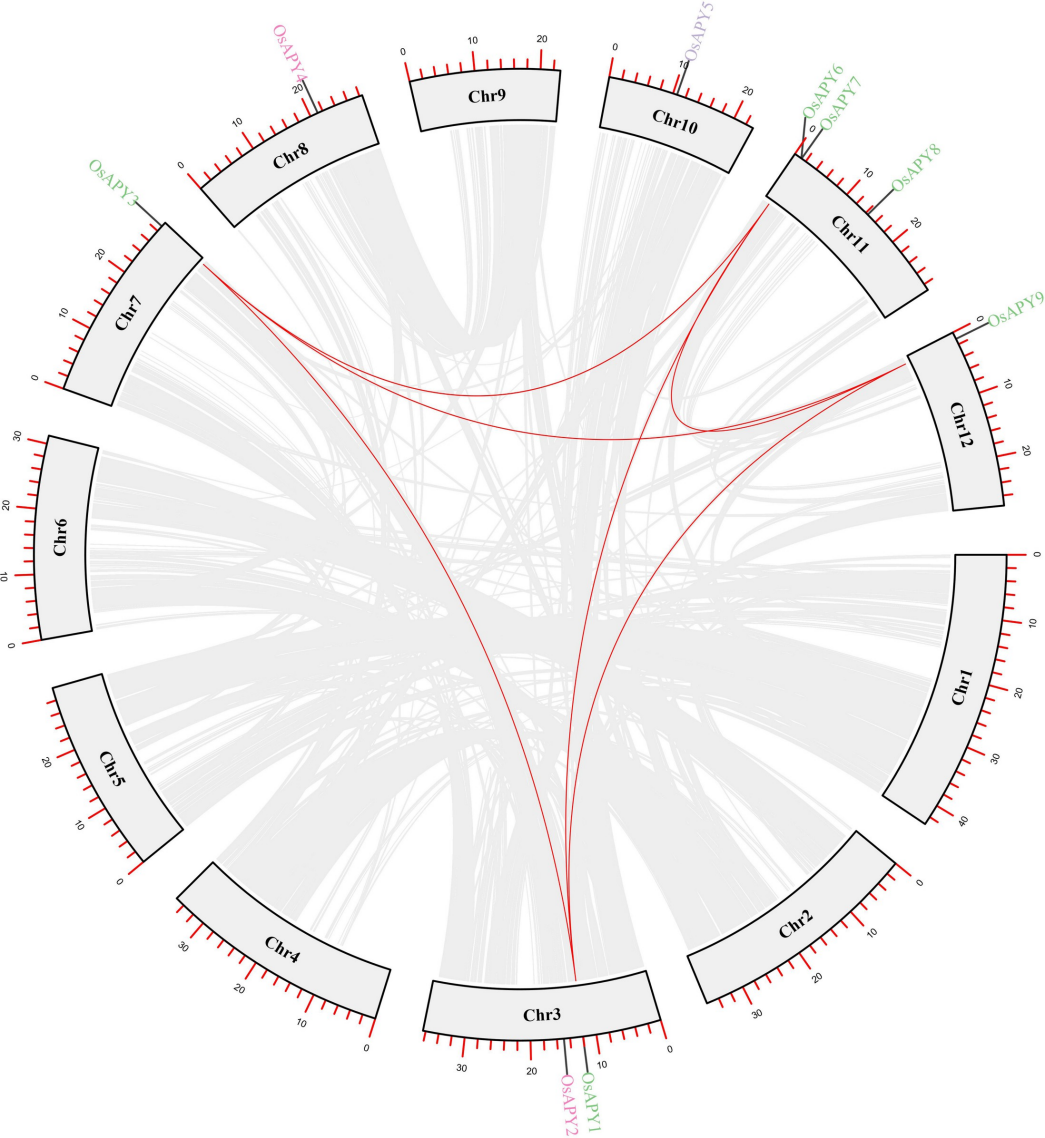
Duplication events of *OsAPY*s within rice genome. Duplication of the *OsAPY*s was specified by the red lines, and the one among the whole rice genome was denoted by the gray lines. TBTools was used to create the circle plot.

Duplication events are crucial for broadening the gene family and for the emergence of novel gene functions. The rice genome had a total of six duplications, three of which were in the *APY* family (Fig 3 and S2 Table). There was no tandem duplication since no genes on the same chromosome had been duplicated. In this study, the duplication type was segmental. The three groups of segmentally duplicated genes within the rice apyrase gene family were *OsAPY1/9*, located on chromosomes 3 and 12, respectively; *OsAPY1/3*, found on chromosomes 3 and 7, respectively; *OsAPY3/9*, situated on chromosomes 7 and 12 respectively.

The non-synonymous (Ka) and synonymous (Ks) substitution rate of genes was used to measure the selection pressure of duplication occurrences. Ka/Ks = 1 implies neutral selection, whereas Ka/Ks<1 represents purifying selection, and Ka/Ks>1 denotes positive selection [96]. Between 0.1476 to 0.5560, Ka/Ks of segmental duplication had a mean of 0.28021. As demonstrated by Ka/Ks ratios lower than 1, all the genes originated under the influence of purifying selection (S2 Table). The duplication time of each event was counted in million years ago (MYA) unit (S2 Table), and it occurred between 66.6 MYA and 1.54 MYA.

Eight orthologous pairs were found between rice *OsAPY*s and other species (Table 2). It was duplicated with sorghum, western poplar, cotton, grapevine, maize, and soybean. The range of Ka/Ks was between 0.143 and 0.357, and the mean was 0.2471. These data suggested that duplication events had a significant impact on the expansion and functional diversity, and an important role was served by segmental duplication in the evolution of the *APY* family of genes.

**Table 2:** Duplication event between rice *APY* genes and other species.

Duplicated Gene 1	Duplicated Gene 2	Ka	Ks	Ka/Ks	Duplication time (MYA)	Purifying Selection	Duplication type
*OsAPY2*	Sobic.001G356200 (Sorghum)	0.1427	0.4936	0.2891	16.45	Yes	Segmental
*OsAPY1*	Potri.019G031000 (Western Poplar)	0.2653	1.6902	0.157	56.34	Yes	Segmental
*OsAPY1*	Gorai.007G268200 (Cotton)	0.3114	1.6822	0.1851	56.07	Yes	Segmental
*OsAPY2*	GRMZM2G056944 (Maize)	0.16	0.4481	0.3571	14.93	Yes	Segmental
*OsAPY2*	GRMZM2G097987 (Maize)	0.158	0.4801	0.3291	16	Yes	Segmental
*OsAPY4*	Glyma.02G107700 (Soybean)	0.4302	1.6258	0.2646	54.19	Yes	Segmental
*OsAPY4*	Glyma.01G047100 (Soybean)	0.4293	1.7026	0.2521	56.75	Yes	Segmental
*OsAPY4*	GSVIVT01019977001 (Grape vine)	0.3133	2.1857	0.1433	72.85	Yes	Segmental

### Cis-acting regulatory elements (CAREs) analysis

**Fig 4:**
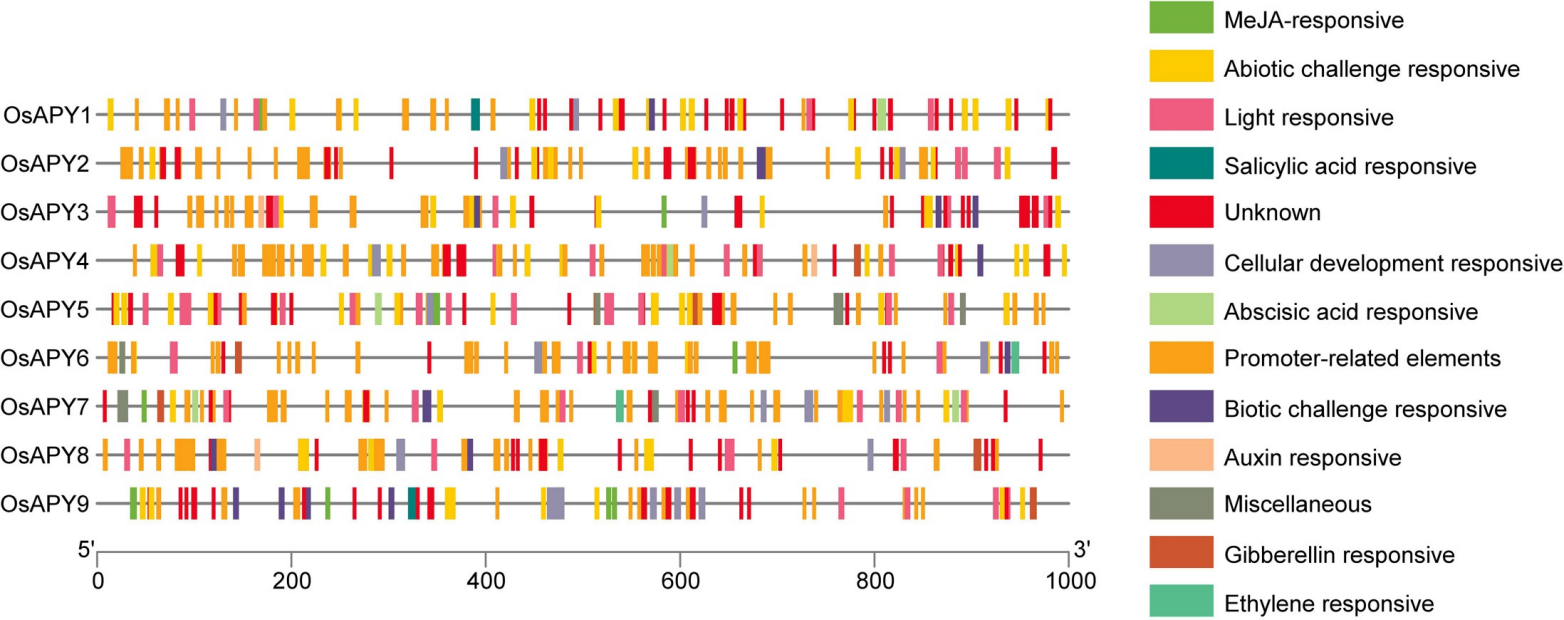
Schematic diagram of predicted cis-elements in *OsAPY* promoter region. Upstream nucleotides of the translation initiation point are shown by the scale at the end. Different colored boxes depict different cis-regulatory elements.

Rice *APY* genes might be regulated by promoter sequences, which are known to be associated with the regulation of transcription of the genes in plants. Analysis of 1 kbp upstream promoter sequences in *O*. *sativa* indicated the presence of 69 CAREs. These cis-elements were grouped into eight different functional categories (Fig 4): i) Light responsive cis-elements, ii) Abiotic challenge responsive elements, iii) Hormonal regulation responsive elements, iv) Elements responsible for cellular development, v) Promoter associated elements, vi) Biotic challenge responsive elements, vii) Elements with miscellaneous functions and viii) Elements with unknown functions. Hormonal regulation responsive elements were further divided into a) MeJA responsive, b) Salicylic acid-responsive, c) Auxin responsive, d) Gibberellin responsive, e) Ethylene responsive, and f) Abscisic acid-responsive elements (Fig 4).

Different abiotic stress-associated elements (anaerobic, anoxic, low temperature, drought, salt, and cold) were observed in the *OsAPY* promoter region (Table 3). STRE, TC-rich repeats, MBS, ARE, GC-motif, LTR, CCAAT-box, AP-1, DRE core, MYB, MYC¸ were the abiotic challenge responsive cis-elements. Among all these elements, STRE was present in the highest frequency, and DRE1 was present in the lowest frequency. W box, WRE3, and WUN-motif (Table 3), the biotic challenge responsive elements, mainly were found to act as fungal elicitors and wound-responsive elements. WRE3 occurred in the highest frequency, and WUN-motif occurred in the lowest frequency.

**Table 3:** List of cis-regulatory elements (CREs) found in the 5′ UTR region of the *OsAPY*s.

Responsive factors	CREs	Function	Reference(s)
Hormonal regulation responsive	ABRE	Involved in the abscisic acid responsiveness	[97]
	ABRE3a	Involved in the abscisic acid responsiveness	[98]
	ABRE4	Involved in the abscisic acid responsiveness	[98]
	GARE-motif	Gibberellin-responsive element	[99, 100]
	P-box	Gibberellin-responsive element	[101]
	TCA	Involved in salicylic acid responsiveness	[23]
	TCA-element	Involved in salicylic acid responsiveness	[101, 102]
	TGA-element	Auxin-responsive element	[103]
	CARE	Enhances the level of gibberellin (GA) ‐induced expression	[104]
	CGTCA-motif	Involved in the Methyl Jasmonate (MeJA)-responsiveness	[103]
	TGACG-motif	Involved in the MeJA-responsiveness	[105]
	ERE	Ethylene-responsive element	[101]
	as-1	Involved in the MeJA-responsiveness	[106]
	box S	Regulate jasmonate‐ and elicitor‐responsive expression	[107]
Cellular development	CAT-box	Related to meristem expression	[108]
	HD-Zip 1	Involved in differentiation of the palisade mesophyll cells	[109]
	circadian	Involved in circadian control	[109, 110]
	RY-element	Involved in seed-specific regulation	[111]
	O2-site	Involved in zein metabolism regulation	[103]
	CCGTCC motif	Meristem specific activation	[112]
	CCGTCC-box	Meristem specific activation	[113]
	AAGAA-motif	Involved in secondary xylem development	[114]
	AC-II	Involved in xylem specific expression.	[115]
Light responsive	3-AF1 binding site	Light responsive element	[99]
	AE-box	Part of a module for light response	[101]
	ATCT-motif	Part of a conserved DNA module involved in light responsiveness	[100]
	Box 4	Part of a conserved DNA module involved in light responsiveness	[116]
	G-Box	Involved in light responsiveness	[101]
	G-box	Involved in light responsiveness	[103]
	GA-motif	Part of a light responsive element	[117]
	GATA-motif	Part of a light responsive element	[118]
	GT1-motif	Light responsive element	[119]
	Gap-box	Part of a light responsive element	[120]
	I-box	Part of a light responsive element	[116, 121]
	L-box	Part of a light responsive element	[118]
	MRE	MYB binding site involved in light responsiveness	[118]
	Sp1	Light responsive element	[118]
	TCCC-motif	Part of a light responsive element	[122]
	TCT-motif	Part of a light responsive element	[123]
Abiotic challenge responsive	AP-1	Stress responsive cis-element	[124]
	ARE	Essential for the anaerobic induction	[115]
	GC-motif	Enhancer-like element involved in anoxic specific inducibility	[124]
	LTR	Involved in low-temperature responsiveness	[103]
	MBS	MYB binding site involved in drought-inducibility	[126]
	TC-rich repeats	Involved in defense and stress responsiveness	[103]
	CCAAT-box	MYBHv1 binding site	[127]
	DRE core	Regulation of drought, high‐salt, and cold stresses	[128]
	MYB	Involved in drought, low temperature, salt and ABA stress responses	[129, 130]
	MYB recognition site	Involved in the drought-induced expression	[129]
	MYC	Involved in early response to drought and abscisic acid induction	[131]
	STRE	Involved in defense and stress responsiveness	[125]
Biotic challenge responsive	W box	Fungal elicitor responsive element	[132]
	WRE3	Wound-responsive element	[125]
	WUN-motif	Wound-responsive element	[132]
Promoter-related elements	CAAT-box	Common cis-acting element in promoter and enhancer regions	[103]
	TATA-box	Core promoter element around -30 of transcription start	[103]
Miscellaneous	3-AF3 binding site	Part of a conserved DNA module array (CMA3)	[133]
	AT-rich sequence	Binding site of AT-rich DNA binding protein (ATBP-1)	[103]
	Box III	Protein binding site	[118]
	Myb-binding site	Myb-binding site	[134]
	MYB-like sequence	MYB recognition sequence	[134]

TGACG motif, CGTCA motif, TGA element, TCA element, GARE-motif, ABRE, P-box, ERE, as-1, and box S constituted the hormonal regulation responsive cis-elements (Table 3). Cellular development-responsive cis-elements played a major role in meristem expression, palisade mesophyll tissue differentiation, circadian control, seed-specific regulation, zein metabolism regulation, and xylem-specific expression (Table 3). These cis-elements included CAT-box, HD-Zip 1, circadian, RY-element, O2-site, CCGTCC motif, CCGTCC box, AAGAA-motif, and AC-II.

Light responsive cis-elements included 3-AF1 binding site, TCT-motif, TCCC-motif, MRE, Gap-box, I-box, GT1-motif, ATCT-motif, Box 4, GATA-motif, GA-motif, G-box, G-Box, AE-box, L-box, Sp1, (Table 3). They mainly functioned as a light-responsive element or a module or a component of such an element. TATA-box and CAAT-box were the two elements, related to the promoter, found in *OsAPY* genes, and they were core promoter elements that mainly functioned in promoter and enhancer regions. Cis-elements with miscellaneous functions included 3-AF3 binding site, MYB-like sequence Myb-binding site, Box III, and AT-rich sequence (Table 3). *OsAPY5* contained a 3-AF3 binding site that was a DNA module array component. The AT-rich DNA binding protein (ATBP-1) utilized a binding site situated on *OsAPY7*, which was located on the AT-rich sequence. On the other hand, Box III was located on *OsAPY7* as a binding site for proteins.

### Analysis of chromosomal distribution

**Fig 5:**
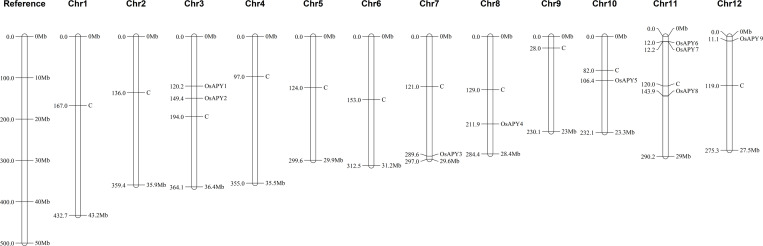
Distribution of *OsAPY*s throughout the rice genome. The figure was generated using MapChart. Chromosome number is presented on top. C depicts the centromere position. The position of every gene can be easily compared following the reference chromosome at the left.

The distribution of APY genes on all 12 of rice’s chromosomes was investigated, and the findings showed that there was an uneven distribution of APY genes (Fig 5). No *OsAPY* member was mapped onto chromosome 1, 2, 4, 5, 6, and 9. It was observed that both *OsAPY1* and *OsAPY2* were on chromosome 3. The chromosomal positions of *OsAPY3*, *4*, *5*, and *9* were 7, 8, 10, and 12, respectively. *OsAPY6-OsAPY8* was located on chromosome 11. Near the centromere, *OsAPY1*, *2*, *5*, and *8* were clustered. *OsAPY6*, *7*, and *9* were situated on the p arm of the chromosome, and on the other hand, *OsAPY3* and *4* were placed on the q arm of the chromosome. The location of all the *OsAPY*s in different chromosomes, their position, and orientation are mentioned in the S3 Table.

### Identification of miRNAs targeting *OsAPY* genes

**Fig 6:**
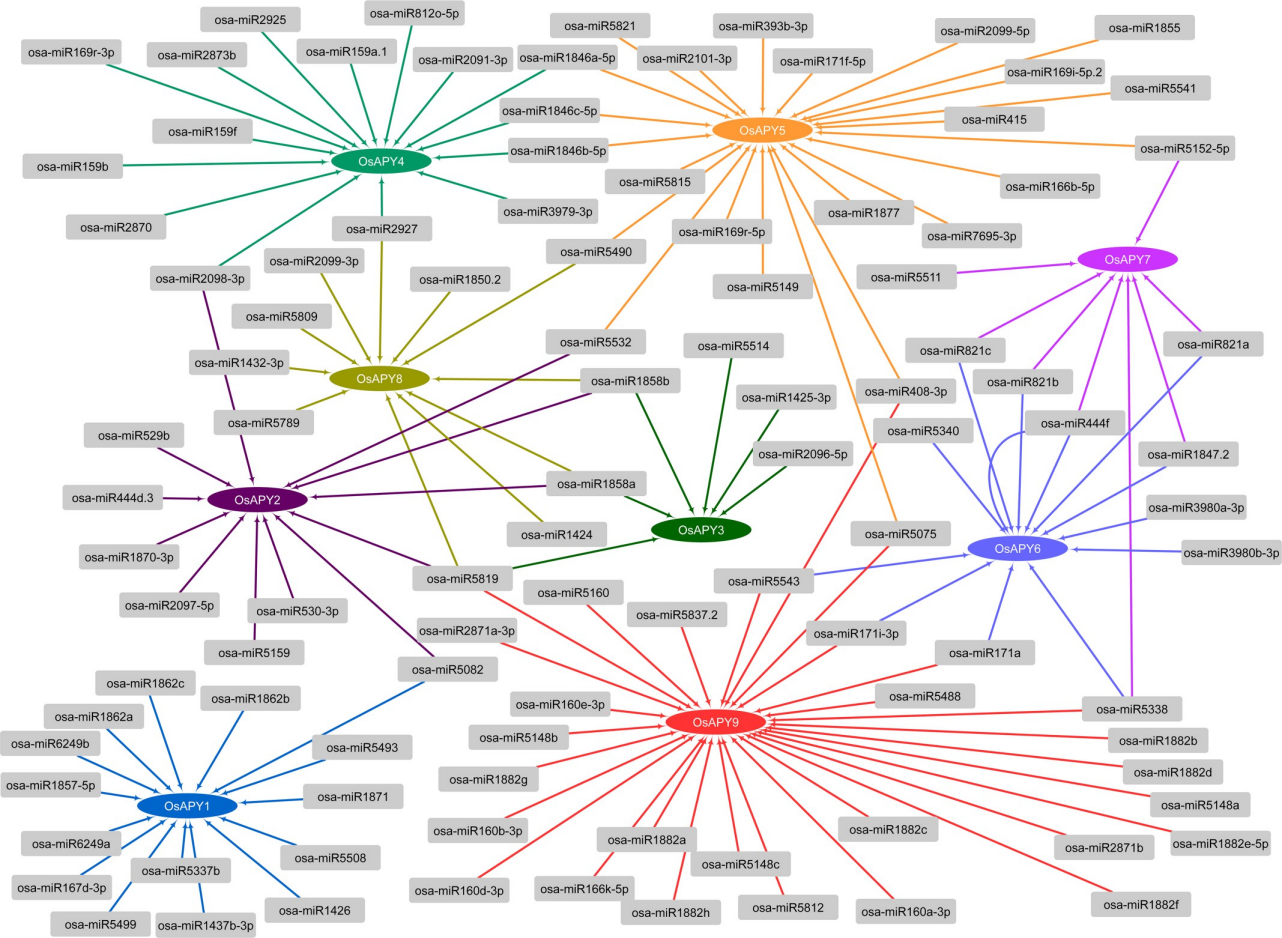
Identification of potential miRNAs targeting *OsAPY* genes. Visual depiction of miRNA-*OsAPY* interaction is generated via Cytoscape. Arrows depict the regulatory relationship, elliptical shapes of different colors represent the *OsAPY*s, and the miRNAs are shown in ash-colored boxes.

In this investigation, 103 putative and distinct rice OsAPY family member-targeting miRNAs with lengths of 19–24 nucleotides were discovered. The highest number of miRNAs targeted *OsAPY9*, whereas *OsAPY3* was targeted by the lowest number. Fig 6 illustrates the regulatory relationships involving potential miRNAs as well as their targeted *APY*s. The majority of the miRNAs expected to target *OsAPY*s had a strong inhibitory effect through cleavage. The inhibitory action for only a few numbers of miRNAs was translation. The list of miRNAs targeting different *OsAPY*s and their mode of inhibition is given in the S4 Table.

**Fig 7:**
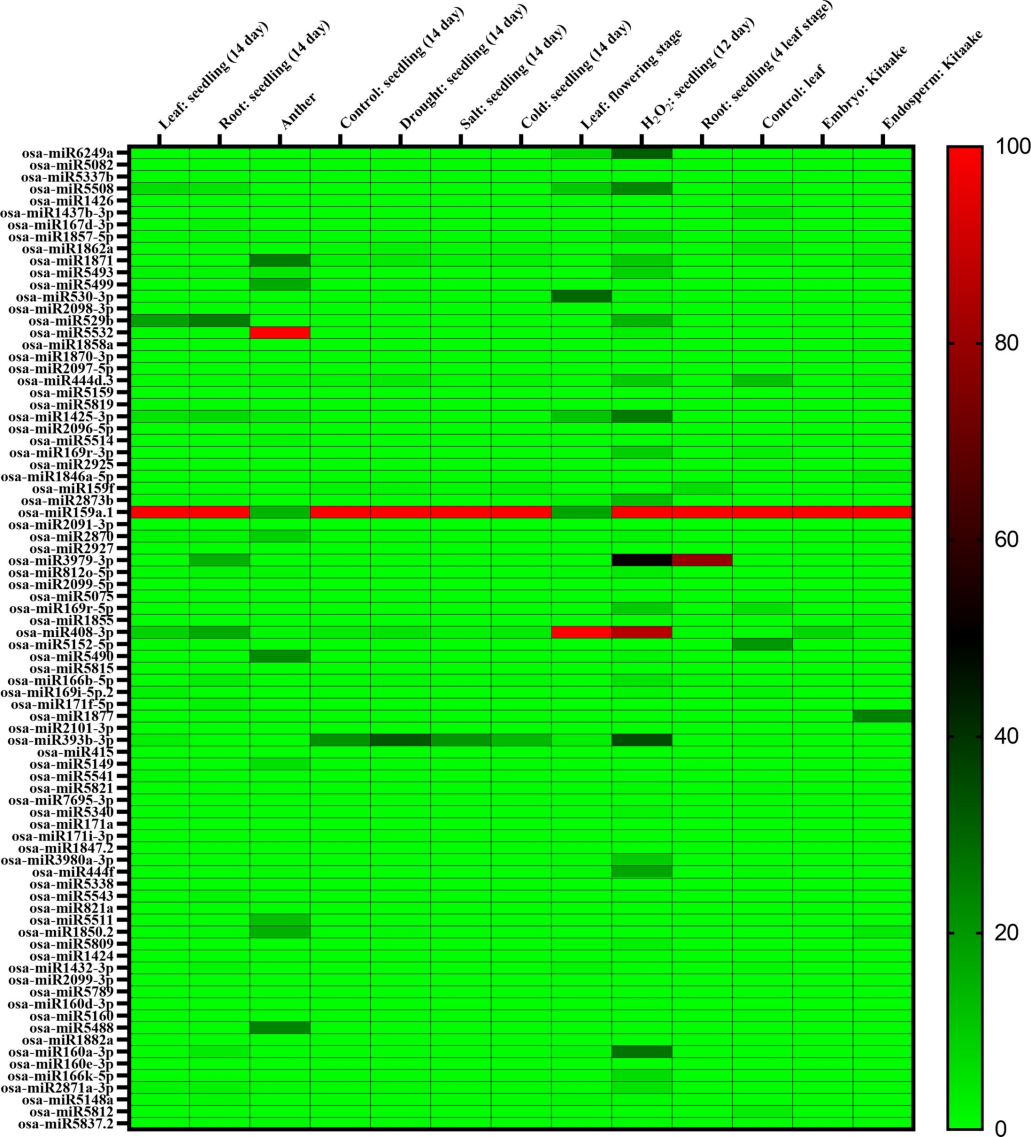
Analysis of *APY*-targeting miRNA expression in different abiotic stresses and tissues. The expression profile of each miRNA can be analyzed following the scale at the right. A heat map was generated using GraphPad Prism 9.0.0.

Twenty-three miRNAs out of all the miRNAs targeted multiple OsAPYs, whereas the remaining miRNAs were specific to each gene. Most miRNAs were found to have lower expression patterns. A heat map was generated (Fig 7) by comparing miRNA expression levels from PmiRExAt [75] across tissues and under a number of different abiotic stress conditions. Among all the miRNAs, osa-miR159a.1 targeting *OsAPY4* was expressed highly in all stresses and tissues except anther and leaves during the flowering stage. The target of osa-miR5532 was *OsAPY2* and *OsAPY5*, which was abundantly expressed in anther. osa-miR3979-3p, which targeted *OsAPY4*, was strongly expressed in roots. osa-miR408-3p targeting *OsAPY5*, *9*, was highly expressed in leaves during the flowering stage and in seedlings exposed to H_2_O_2_. These expressions of most of the miRNAs were downregulated during different stresses suggesting the increased expression of the *OsAPY*s in such stress conditions. Therefore, sequence-specific miRNA-mediated interaction may have a vital function in regulating *OsAPY* genes, which in turn may help plants act on environmental as well as growth signals.

### Study of SNPs in *OsAPY*s

Rice SNP-Seek database [76] was used to better understand the variations in alleles of *OsAPY* members throughout 11 rice varieties and were selected based on their stress responses. It allowed identifying the Single Amino acid Polymorphisms (SAPs) (Table 4). SAPs were identified in 6 *OsAPY*s, but not in *OsAPY1*, *5*, and *8*. It indicated that the genes of *OsAPY1*, *5*, and *8* are substantially conserved among rice genotypes. The indica varieties included NERICA-L-27, Pokkali, Rasi, Vandana, Swarna, and IR-64, while Azucena and NERICA-1 were tropical japonica varieties. GIZA 159, Nagina 22, and Pusa (Basmati 1) were the members of the temperate japonica, aus, and unassigned variety, respectively. The study revealed that indica rice varieties had a higher rate of SAPs than other rice varieties. 5 SAPs were identified in *OsAPY2*; *OsAPY3*, *7*, and *9* showed 3 SAPs, while *OsAPY4* had only one SAP, but *OsAPY6* contained 4 SAPs.

**Table 4:** Distribution of single amino acid polymorphisms (SAPs) throughout the *APY*s of chosen rice varieties.

Gene Name	Chromosomal Position	Nipponbare	Pokkali	Rasi	IR-64	NERICA-L-27	Pusa (Basmati 1)	Swarna	Vandana	NERICA-1	Nagina 22	Azucena	GIZA 159
*OsAPY2*	14938900 (C/T)	A_109_	A_109_ Heat	A_109_	A_109_ Blast Fungus	A_109_	A_109_	V_109_	A_109_ Heat, Blast Fungus	A_109_	A_109_ Gall Midge Pest, Salt	A_109_ Salt	A_109_
	14940235 (G/A)	R_258_	R_258_ Brown Plant hopper Pest	R_258_	R_258_	R_258_	R_258_	R_258_	R_258_	R_258_	R_258_ Bacteria Rice Plant hopper, White-backed Plant hopper Pest, Salt	R_258_ Cold, Salt	H_258_
	14940827 (A/T)	K_295_	K_295_ Heat, Brown Plant hopper Pest	K_295_	K_295_ Blast Fungus	K_295_	K_295_	K_295_	K_295_ Heat	K_295_	N_295_ Salt, White-backed Plant hopper Pest, Bacteria Rice Plant hopper	K_295_ Cold	K_295_
	14941519 (T/G)	S_366_	A_366_ Heat	A_366_	S_366_	S_366_	S_366_	A_366_	A_366_ Heat	A_366_	S_366_ White-backed Plant hopper Pest, Gall Midge Pest	S_366_	S_366_
	14941585 (A/C)	K_388_	Q_388_	Q_388_	Q_388_	Q_388_	Q_388_	Q_388_	Q_388_	Q_388_	Q_388_	K_388_	K_388_
	14941823 (C/T)	T_434_ Bacteria Leaf Blight	T_434_ Heat, Brown Plant hopper Pest	T_434_	I_434_	I_434_	I_434_	T_434_	T_434_ Heat	T_434_	I_434_ Bacteria Rice Plant hopper, White-backed Plant hopper Pest, Salt	T_434_	T_434_
*OsAPY3*	28963897 (A/C)	E_111_	A_111_	A_111_	A_111_	A_111_	A_111_	A_111_	A_111_	E_111_	A_111_	-	E_111_
	28963907 (T/G)	N_114_	K_114_	K_114_	K_114_	K_114_	K_114_	K_114_	K_114_	N_114_	K_114_ Bacteria Rice Plant hopper, White-backed Plant hopper Pest	N_114_	N_114_
	28963972 (T/C)	V_136_	A_136_	A_136_	A_136_	A_136_	A_136_	A_136_	A_136_	V_136_	A_136_	V_136_	V_136_
*OsAPY4*	21193570 (G/T)	R_453_	M_453_	M_453_	M_453_	M_453_	M_453_	M_453_	M_453_	M_453_ R_453_	G_207_	G_207_	R_453_
*OsAPY6*	1202969 (T/A)	M_77_	K_77_	K_77_	K_77_	K_77_	K_77_	K_77_	K_77_	M_77_	-	M_77_	M_77_
	1204513 (T/G)	I_162_	R_162_	R_162_	R_162_	R_162_	R_162_	R_162_	R_162_	I_162_	R_162_ White-backed Plant hopper Pest	I_162_	I_162_
	1204862 (C/T)	P_252_	S_252_	S_252_	S_252_	-	S_252_	S_252_	S_252_	P_252_	S_252_	P_252_	P_252_
	1204889 (G/A)	E_261_	K_261_	K_261_	K_261_	K_261_	K_261_	K_261_	K_261_	E_261_	K_261_	E_261_	E_261_
	1206220 (C/A)	S_457_	Y_457_	Y_457_	Y_457_	Y_457_	Y_457_	Y_457_	Y_457_	S_457_	-	S_457_	S_457_
*OsAPY7*	1224875 (T/C)	I_236_	T_236_	T_236_	T_236_	T_236_	T_236_	T_236_	T_236_	I_236_	T_236_	I_236_	I_236_
	1224893 (A/G)	E_242_	G_242_	G_242_	G_242_	G_242_	G_242_	G_242_	G_242_	E_242_	G_242_	E_242_	E_242_
	1226429 (G/A)	V_448_ Bacteria Leaf Blight	V_448_ Heat	V_448_	V_448_ Blast Fungus	V_448_	V_448_	V_448_	V_448_ Heat	I_448_	V_448_ White-backed Plant hopper Pest	V_448_	V_448_
*OsAPY9*	1107655 (A/G)	N_75_	N_75_ Brown Plant hopper Pest	D_75_	N_75_	N_75_	N_75_	N_75_	N_75_ Heat	N_75_	D_75_ Salt, White-backed Plant hopper Pest, Bacteria Rice Plant hopper	N_75_ Salt	N_75_
	1109364 (A/C)	Q_290_	H_290_	Q_290_	H_290_	H_290_	H_290_	H_290_	H_290_	H_290_	Q_290_ Gall Midge Pest, White-backed Plant hopper Pest, Bacteria Rice Plant hopper	H_290_	Q_290_
	1109753 (A/G)	N_350_	N_350_ Heat, Brown Plant hopper Pest	D_350_	N_350_	N_350_	N_350_	N_350_	N_350_	N_350_	D_350_ Bacteria Rice Planthopper	N_350_ Cold	N_350_

- no polymorphism

Analysis showed that SNPs located at each position were specific for different stresses in particular rice varieties (Table 4). The presence of such stress-resistant SNPs was involved in providing resistance to various stresses in rice varieties. Azucena, a moderately salt, and cold-resistant variety and at the same time susceptible to heat stress [79], contained SNPs that were involved in salt and cold resistance. Similarly, Pokkali was resistant to heat and brown planthopper pest and susceptible to cold and salt stress [79]. The SNPs present in Pokkali were heat and brown planthopper pest resistant and so they can act as markers for these two stress conditions in Pokkali. IR-64 had the SNPs that were blast fungus resistant; Nipponbare had the ones against bacteria leaf blight; Vandana was resistant to heat and blast fungus. As a result, those SNPs can act as markers for screening the putative rice genotypes for that specific stress conditions.

### Analysis of secondary and tertiary structure

**Fig 8:**
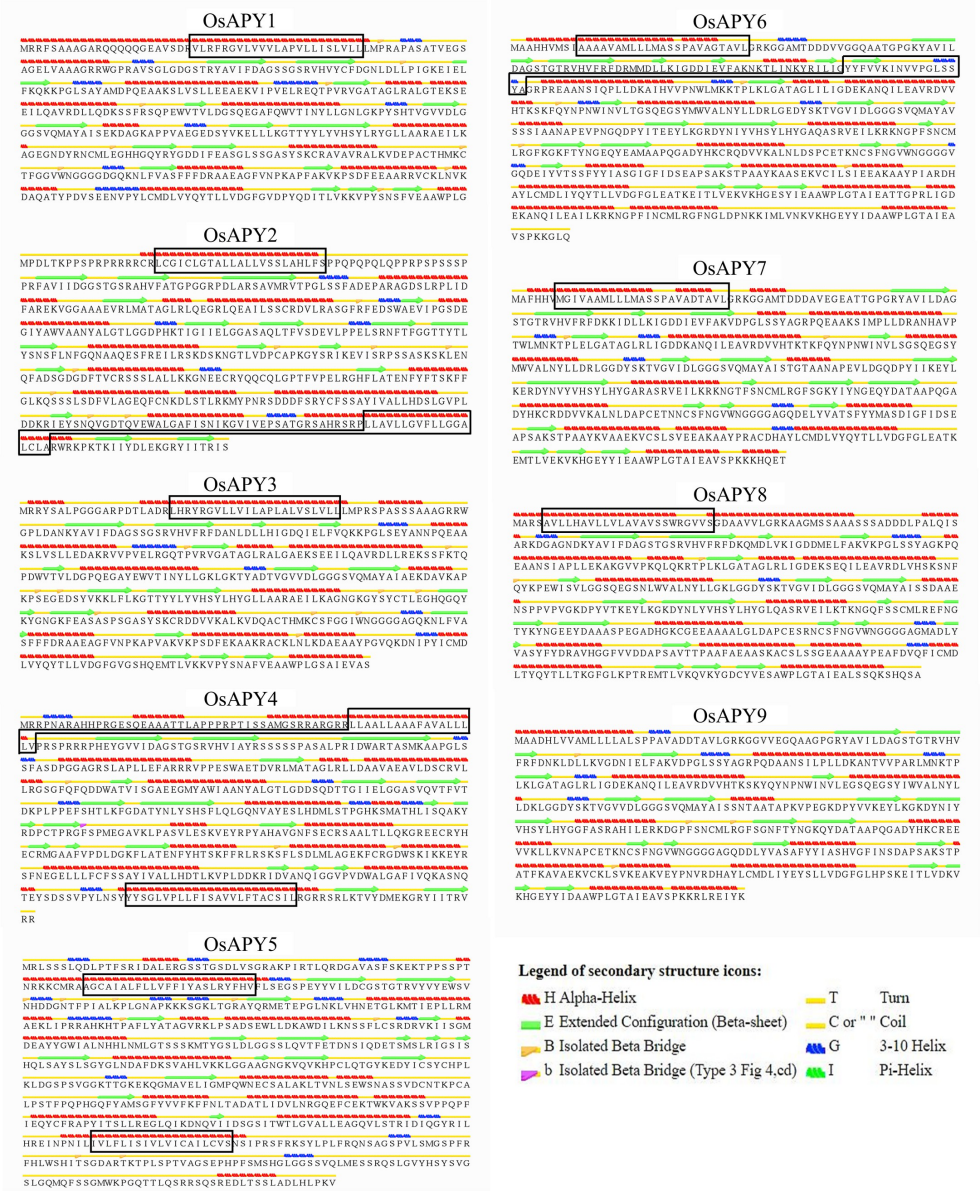
Secondary structure analysis of the nine rice APYs. Secondary structures were generated using the STRIDE program, and the corresponding positions of membrane-spanning motifs were identified via the TMHMM server. The cross-membrane domains were marked with a black colored box. Legend at the bottom-right side shows the icon for each secondary structure.

The secondary structure analysis revealed the position of alpha-helix, beta-sheet, isolated beta bridge, turn, coil, 310-helix, and transmembrane helix (Fig 8). Their percentage and the position of the transmembrane helix are given in the S5 Table. It showed that alpha-helix was dominant over all the other secondary structures, followed by the turn, beta-sheet, coil, and 310-helix. There were some exceptions in OsAPY2, 4, 5, and 8. In OsAPY5, the coil percentage was higher than the beta-sheet, and in OsAPY2, it was higher than the turn and beta-sheet. In OsAPY4 and OsAPY8, the beta-sheet percentage was higher than that of turn. The higher percentage of helix and beta-sheet indicate the stability of the OsAPYs [135], and the structures like random coils are critical for the signaling cascades [136]. The analysis of the motifs spanning the membrane (MSM) indicated that 4 OsAPYs contained one membrane-spanning motif (MSM), 4 OsAPYs contained 2 MSMs, and 1 OsAPY contained no MSM. OsAPY1, 3, 7, 8 contained only one membrane-spanning motif at N-terminal. OsAPY2, 4, 5, and 6 had two MSMs positioned at the N- and C-terminals separately except OsAPY6, where both the MSMs were located on N-terminal. On the other hand, OsAPY9 contained no MSM.

**Fig 9:**
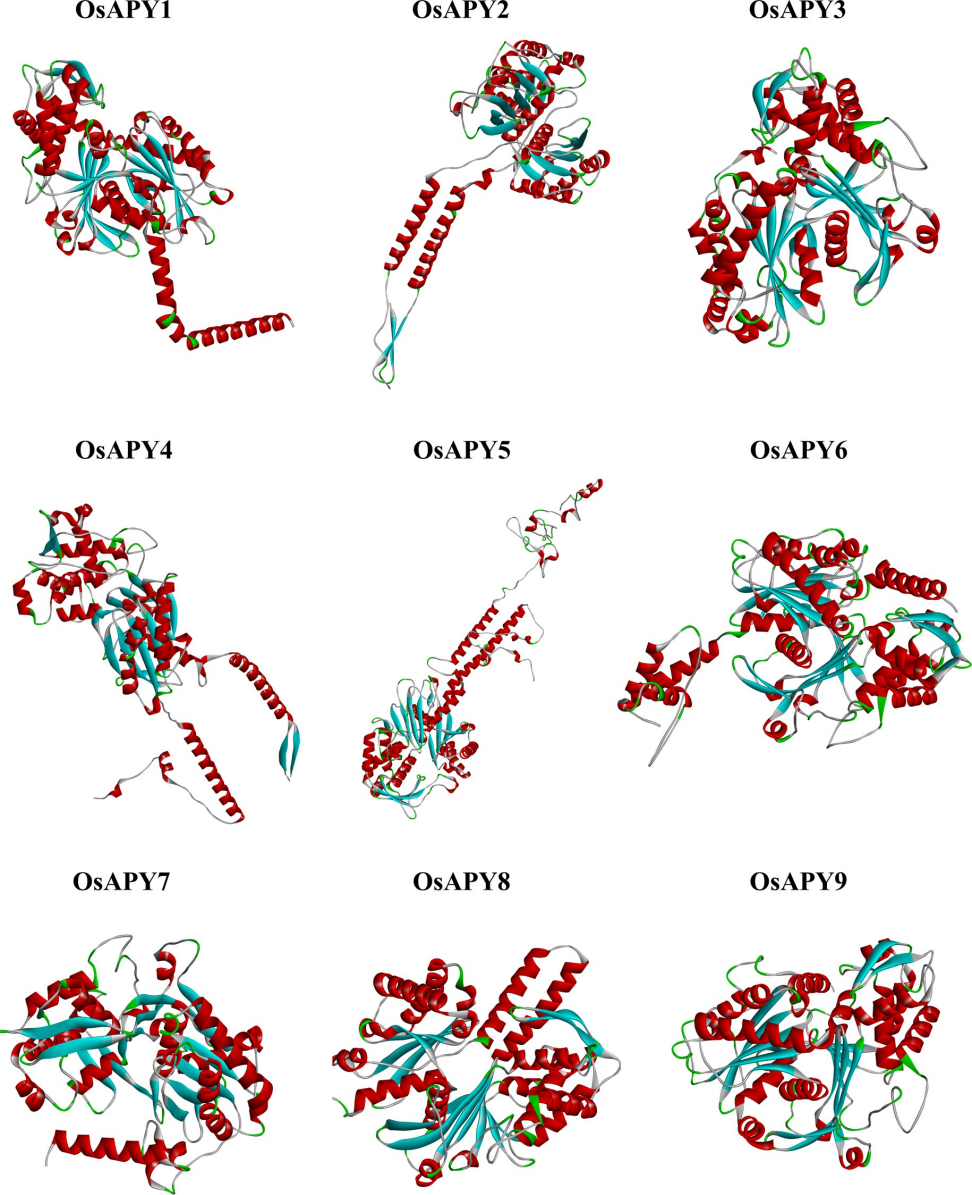
Tertiary structure analysis of the nine rice APYs. The online tool Robetta was used to construct the tertiary structure, and these structures were visualized via Biovia Discovery Studio Visualizer.

RoseTTAFold, a deep learning-based modeling approach, was applied to construct 3D models of proteins using the Robetta web server [82]. The generated models were further refined by GalaxyRefine [83], and then the energy minimization was done via Swiss-PdbViewer [84]. Fig 9 illustrates the modeled tertiary structures of all the OsAPY proteins, visualized via Discovery Studio. These generated structures were then subjected to validation analysis utilizing PROCHECK [85], ERRAT [86], and ProSA-web server [87] (S6 Table). Ramachandran plot analysis in PROCHECK was used to evaluate protein quality [85]. In the favored and additional allowed regions, over 90% of the residues were located, with only <1.5% in the disallowed regions, which confirmed that the projected models were of good quality (S2 Fig). The proteins exhibited an overall quality factor of >87, according to ERRAT [86] analysis (S3 Fig). ProSA-web [87] revealed the Z-score, the level of nativeness of the designed models (S6 Table), and the energy plot, which showed the local quality of the models. The Z-score was found to be in the spectrum of values generally reported for native proteins, implying higher quality of the structures generated, and the models were well within the range of X-ray crystal structure (S4 Fig). As per the energy plot (S5 Fig), all the residues in the simulated structure had a lower value of energy. These findings indicated the excellent quality of the tertiary structures of modeled proteins.

### Analysis of molecular docking

**Fig 10:**
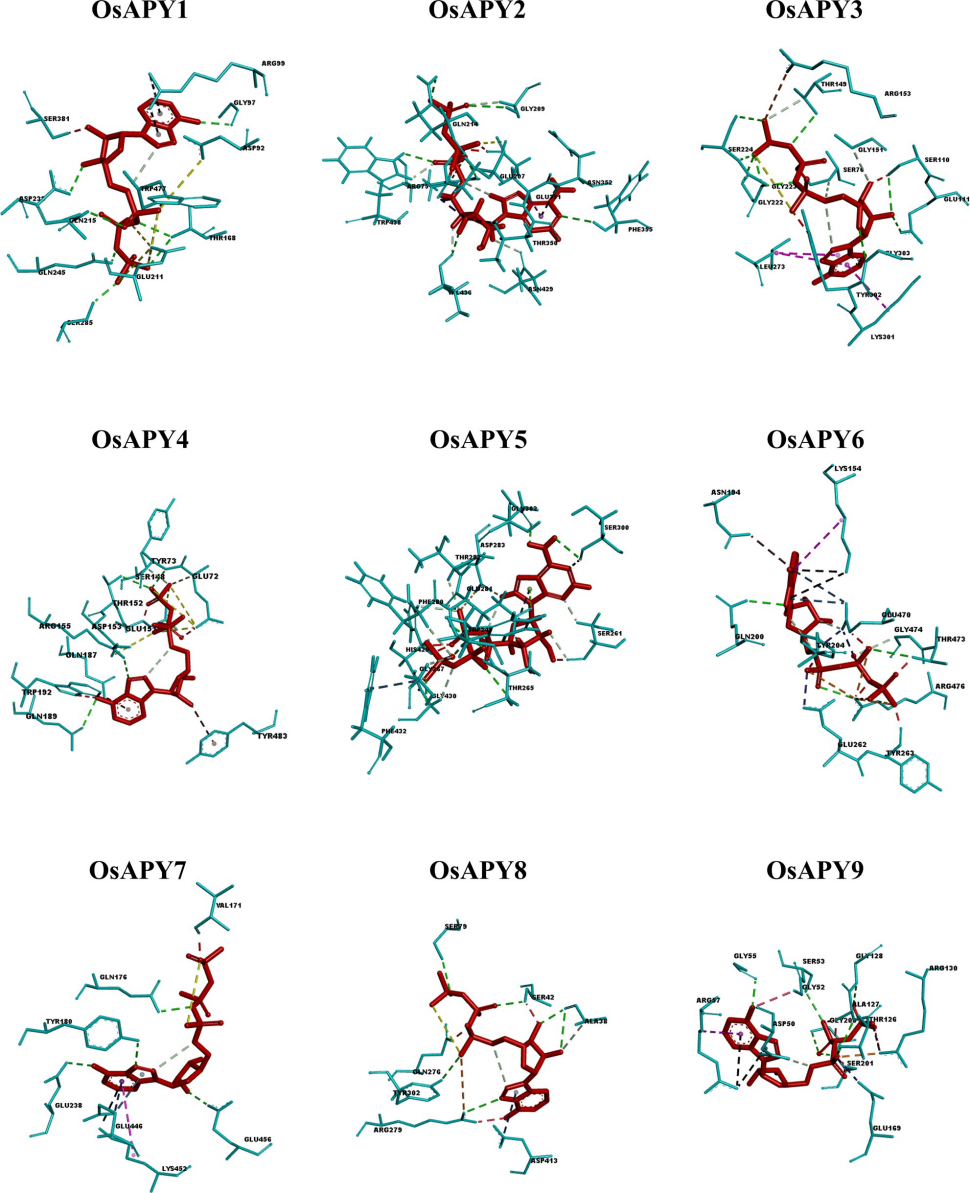
Protein-ligand residual interaction analysis of OsAPYs with ATP depicted by aqua and red color, respectively. Illustration of bonds: carbon-hydrogen bonds- snowy mint lines, conventional hydrogen bonds- green lines, pi-cation bonds- black lines, unfavorable bumps- red lines, unfavorable negative-negative interactions- yellow lines, attractive charge interactions- orange lines, pi-donor hydrogen bonds- deep brown lines, pi-sigma bonds- light purple lines, unfavorable acceptor- acceptor interactions- navy blue lines, pi-alkyl bonds- magenta lines, unfavorable donor-donor interactions- pink lines, pi-anion bonds- deep-sea blue lines, pi-lone pair interactions- lemon green lines, salt bridge- purple lines.

ATP, a vital ligand of APY proteins, was chosen for the analysis of docked protein-ligand complex. The docking analysis was done using the HDOCK server, and it revealed that the docking score of the protein and ATP was between -199.05 and -152.53 (S7 Table). This optimal docking indicated an excellent binding affinity. According to PDBSum [90] analysis, the protein and ATP complex contained 3 to 11 hydrogen bonds (S8 Table). The interaction between the protein and ATP visualized via Discovery Studio [88] revealed the presence of different types of H-bond, electrostatic bonds, and hydrophobic bonds (Fig 10 and S9 Table). Validation of the protein-ligand complex via PROCHECK and ERRAT suggested the high quality of the complexes (S6 and S7 Figs).

OsAPY1 formed seven conventional hydrogen bonds, 2 Pi-cation interactions, and seven unfavorable interactions (Fig 10). Among all the protein residues, THR168 formed 2 of the seven hydrogen bonds, and ARG99 formed both the Pi-cation interactions (S9 Table). OsAPY2 had one attractive charge interaction, 1 Pi-donor hydrogen bond, six conventional hydrogen bonds, five unfavorable interactions, six carbon-hydrogen bonds, and 1 Pi-sigma interaction with ATP. OsAPY3 made one attractive charge interaction, three Pi-alkyl bonds, carbon-hydrogen bonds, two unfavorable interactions, and nine conventional hydrogen bonds. OsAPY4 created four conventional hydrogen bonds, 2 Pi-donor hydrogen bonds, and two carbon-hydrogen bonds. OsAPY5 formed ten carbon-hydrogen bonds, six conventional hydrogen bonds, 1 Pi-lone pair interaction, 2 Pi-anion interactions, and seven unfavorable interactions. OsAPY6 made 2 Pi-cation interactions, Pi-donor hydrogen bonds, carbon-hydrogen bonds, Pi-anion interactions, four conventional hydrogen bonds, four attractive charge interactions, and 1 Pi-alkyl interaction. OsAPY7 and OsAPY8 formed 9 and 12 interactions with ATP, respectively. OsAPY9 had one salt bridge interaction, two attractive charge interactions, seven conventional hydrogen bonds, 2 Pi-cation interactions, 1 Pi-anion interaction, 1 Pi-alkyl interaction, and eight unfavorable interactions. These bonds between the OsAPYs and ATP pointed to a robust docking interaction.

### Investigation of the expression profiles of *OsAPY* genes using RNA-seq data

**Fig 11:**
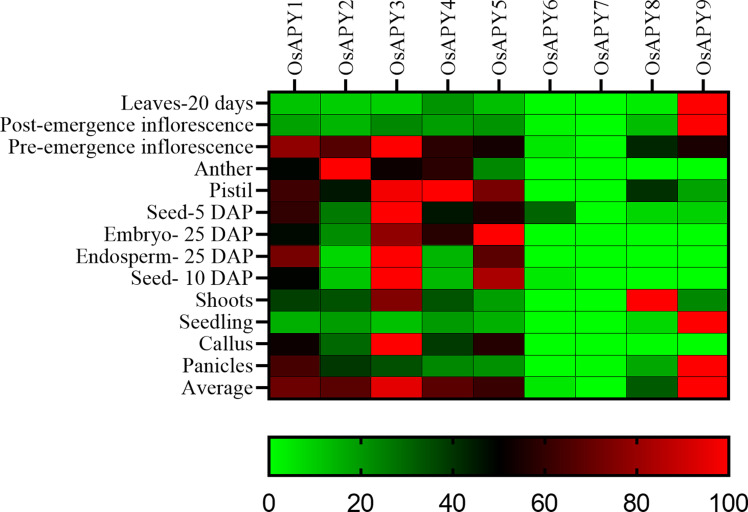
Heatmap of the expression pattern of the *OsAPY* genes according to tissue type. The RNA-seq data collected from the Rice Genome Annotation Project was used to conduct expression analysis. The heat map was generated using GraphPad Prism 9.0.0. Expression profile can be analyzed following the bottom located scale. The red boxes represent high expression rates, greens depict low expression rates, and black boxes signify moderate expression levels.

Using RNA transcript profiling to analyze gene expression is an efficient technique. To better comprehend the expression profile of *OsAPY*s, the RNA-seq data was utilized under several tissue types and stresses (Fig 11). The Rice Genome Annotation Project [52] provided RNA-seq data for *OsAPY* expression profiling in several tissues which was used to produce a heatmap (Fig 11).

The analysis revealed that the expression was higher in the inflorescence, seed, pistil, embryo, and endosperm. *OsAPY1* and *3* were upregulated in most of these tissues, whereas in the pre-emergence inflorescence and anther, *OsAPY2* and *4* were upregulated. *OsAPY4* was expressed highly in the pistil. For *OsAPY5*, it was higher in pistil, seed, embryo, and endosperm among all these tissues. Some of these showed an expression pattern that was tissue-specific, including *OsAPY2*, upregulated in anther, inflorescence, and downregulated in the other types of tissues. In the same manner, the expression of *OsAPY8* was high in shoots. All the *OsAPY*s were downregulated in leaves, post-emergence inflorescence, and seedlings except *OsAPY9*, which was upregulated in these tissues. In shoot tissue, *OsAPY*3 and *8* were highly expressed; on the other hand, *OsAPY3* and *5* were upregulated in callus, and it was *OsAPY1* and *9* in panicles.

**Fig 12:**
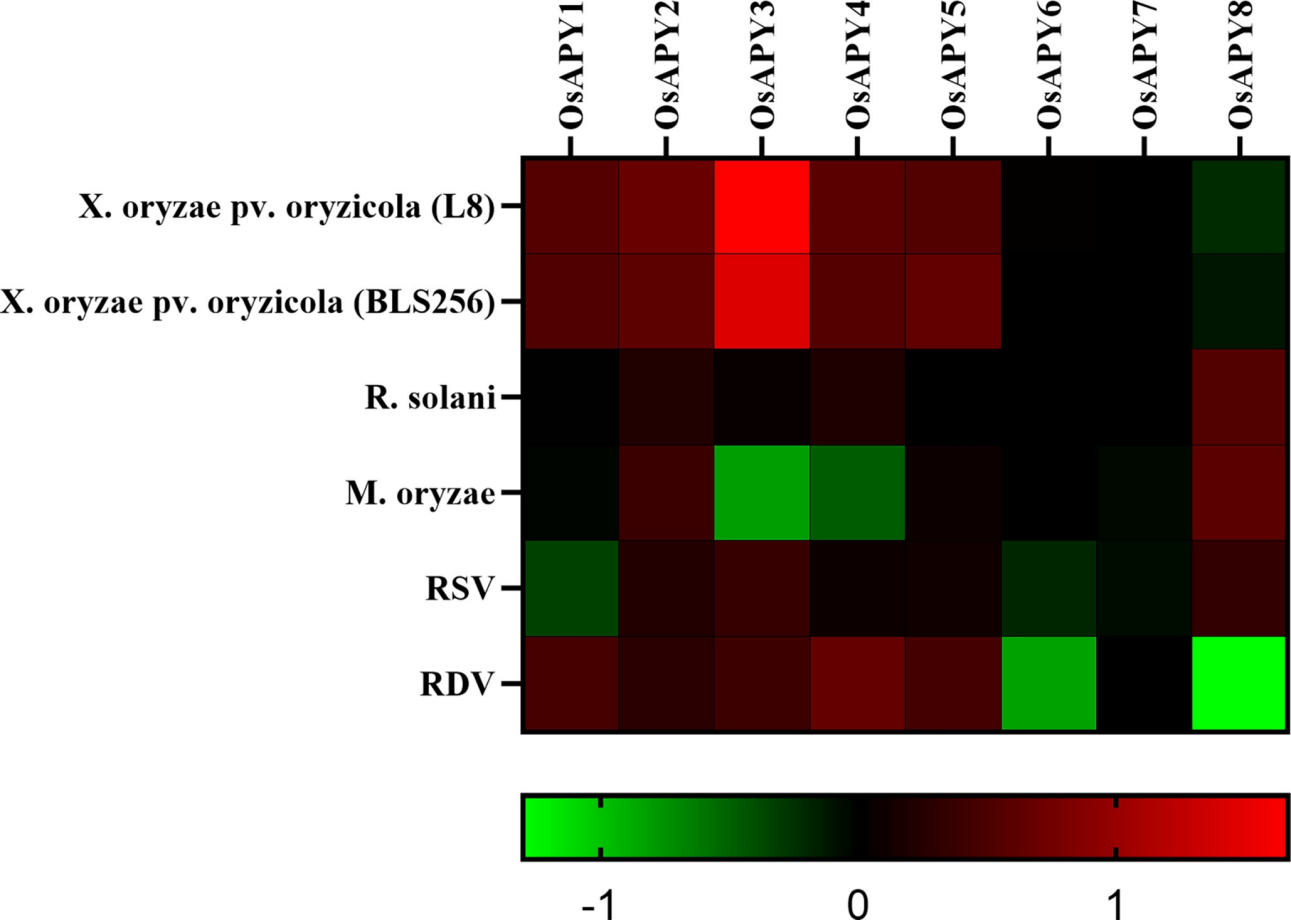
Heatmap of *OsAPY* expression profile in response to various biotic stress conditions. Expression analysis was done utilizing the RNA-seq values of relative expression from GENEVESTIGATOR and using GraphPad Prism 9.0.0, the heat map was generated. The expression profile could be analyzed according to the scale just at the base. The red boxes represent upregulated expression; greens depict downregulated expression, and black boxes signify no change in expression levels.

**Fig 13:**
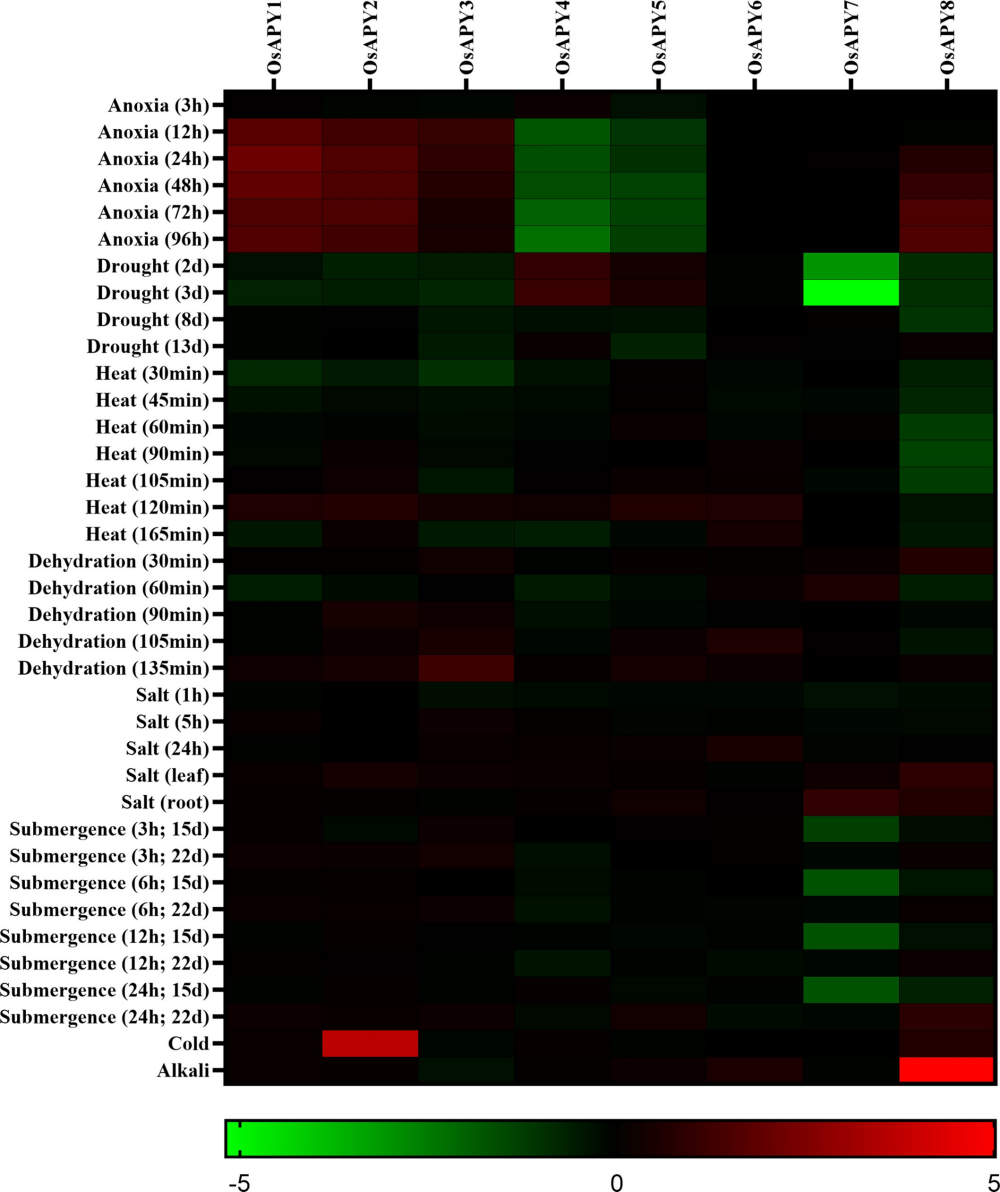
Heatmap of *OsAPY* expression profile in response to various abiotic stress conditions. Expression analysis was done using the RNA-seq values of relative expression from GENEVESTIGATOR. The heat map was generated using GraphPad Prism 9.0.0. The expression profile could be analyzed according to the scale just at the base. The red boxes represent upregulated expression; greens depict downregulated expression, and black boxes signify no change in expression levels.

The profile of expression from RNA-seq data of *OsAPY*s was investigated to reveal their role in response to different stresses. Their expression profile revealed expression in abiotic and biotic stresses. Figs 12 and 13 show a heat map of their expression profile in biotic and abiotic stress. There was no pre-analyzed RNA-seq data of *OsAPY9*, so the gene expression data of only eight genes were analyzed.

In response to infection by two strains of rice bacterial leaf streak pathogen (*Xanthomonas oryzae* pv. *oryzicola)*, *OsAPY1-5* were upregulated, but *OsAPY8* was downregulated (Fig 12). This finding addressed the role of *OsAPY*s in managing rice stress responses against bacterial leaf streak pathogens. It was observed that the infection with *Rhizoctonia solani* upregulated the expression of *OsAPY2*, *4*, and *8*. Rice blast fungus (*Magnaporthe oryzae*) inoculation induced the expression of *OsAPY2*, *5*, and *8* indicating their possible role in rice blast disease. Infection with rice dwarf virus (RDV) upregulated the expression of *OsAPY1-5*; on the other hand, *OsAPY6*, *8* were downregulated. *OsAPY2*, *3*, *5*, and *8* got upregulated in response to rice stripe virus (RSV) infection, but at the same time, *OsAPY1* and *6* got downregulated.

During anoxic conditions, the expression of *OsAPY1-3* and *8* was upregulated, but *OsAPY4* and *5* were downregulated (Fig 13). *OsAPY4* showed a slight increase in expression only in 3 hours-long anoxic conditions. In *OsAPY8*, the level of expression was proportional to the duration of the anoxic condition. Rice subjected to drought for 2 and 3 days showed upregulation of *OsAPY4* and *5*, but all the other *OsAPY*s, especially *OsAPY7*, were downregulated except *OsAPY6*, which showed no change in expression. The extended period of drought condition downregulated the expression level. The gene expression level increased with time duration in heat stress, but after a specific time, it started to decrease. In the case of heat treatment for 120 minutes, the expression of *OsAPY1-6* was upregulated, but gene expression levels were reduced during an extended period of heat treatment (165 minutes). This result indicated that *OsAPY*s displayed late-term responses to heat stress, but extended stress interrupted their expression. When subjected to dehydration, similar late-term expression was observed. 135 minutes long dehydration stress induced the expression of all the *OsAPY*s except *OsAPY4* and *7*. Under salt stress, the expression was relatively higher in leaves than in roots. All the genes except *OsAPY1*, *5*, and *6* were upregulated in leaves, whereas in root tissues, only *OsAPY5*, *7*, and *8* were upregulated. Under submergence stress, the expression level increased with the increase in time. As the heatmap depicted (Fig 13), after 22 days of submergence for 24 hours, there was an increase in the expression of *OsAPY1*, *3*, *5*, and *8*. *OsAPY5* and *8* had an increased expression after prolonged exposure to submerged conditions as there was no significant expression in the case of short-term exposure to submerged conditions. Alkali treatment upregulated the expression of *OsAPY5*, *6*, and especially of *8*, whereas, for *OsAPY2* and *8*, the expression was induced in cold.

### Investigation of expression profile using RT-qPCR data in response to several abiotic stresses

**Fig 14:**
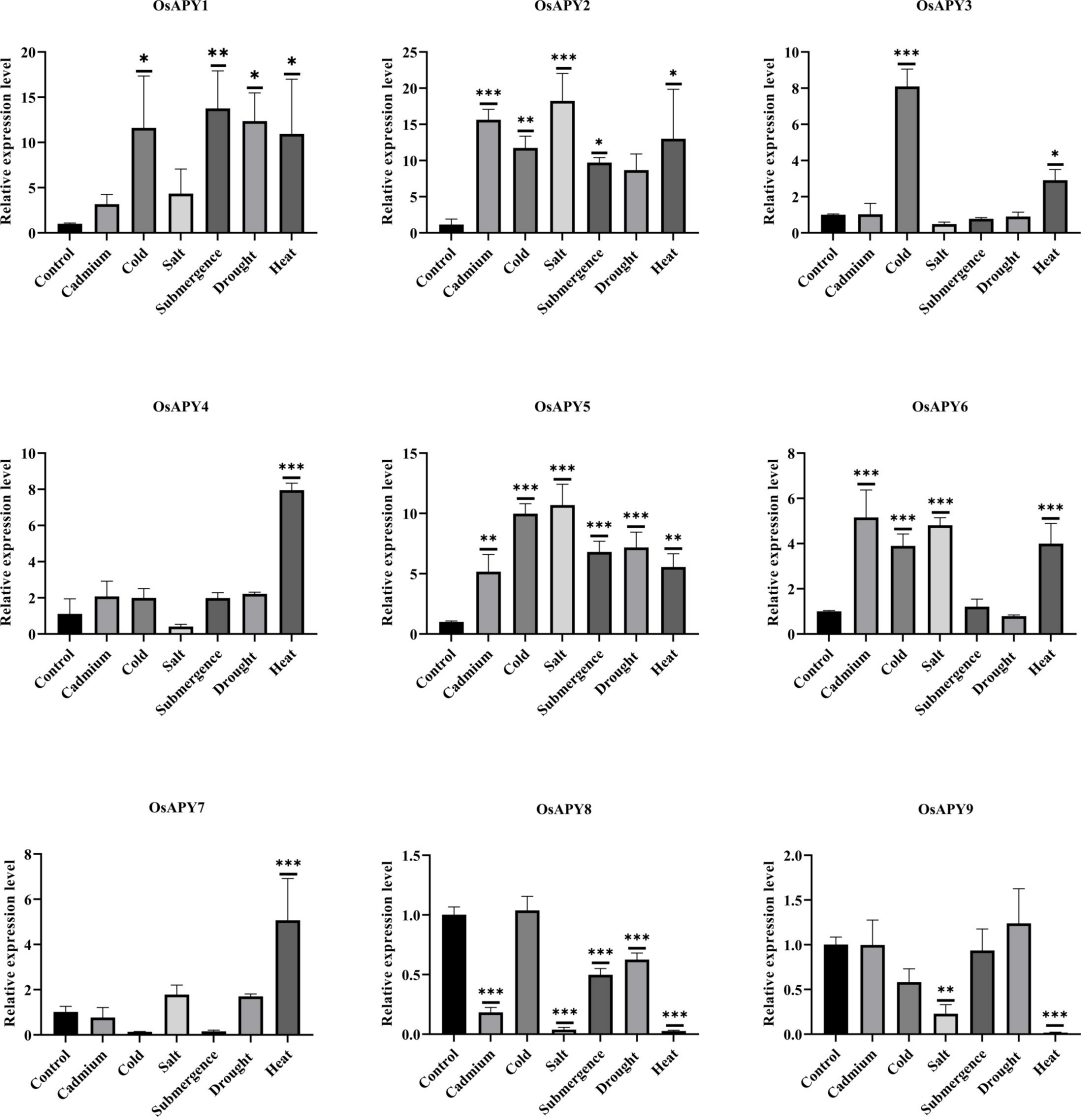
Analysis of relative expression of *OsAPY*s under various abiotic stresses. The Y-axis depicts the relative expression of each gene analyzed via RT-qPCR in the rice plant leaves. The X-axis indicates the various abiotic stress conditions under which the relative expression analysis was done. Technical replication was used to find the mean value of expression at different treatments. MS Office 365 and GraphPad Prism 9.0.0 were used to analyze the data. A one-way ANOVA, followed by a Bonferroni post hoc test, was used to assess the significant differences (P ≤ 0.05). To represent the significant differences, different means were labeled with the different number of asterisks (*). *, **, and *** represent the various level of significance (P ≤ 0.05, P ≤ 0.01, P ≤ 0.001 respectively).

The relative expression ratio of *OsAPY* genes in the rice seedling leaves was assessed under cold, cadmium, salinity, submergence, drought, and heat stress. A bar chart of their real-time expression data in these different stress conditions after 18–20 hours is depicted in Fig 14.

As shown in Fig 14, all the *OsAPY* genes except *OsAPY8* and *9* showed significantly higher expression under heat stress. Among all the stress environments, *OsAPY5* had considerably higher expression levels (more than five-fold) in all the stress conditions. The RNA-seq data (Fig 13) also demonstrated its enhanced expression during drought, submergence, dehydration, alkali, salt, and heat stress.

Cold, submergence, drought, and heat stress, all resulted in considerably more significant levels of *OsAPY1* expression. The expression level during submzergence stress (more than ten-fold) was significantly higher, and the result obtained from RNA-seq data (Fig 13) corroborated this observation. Under cadmium, cold, salinity, submergence, and heat stress, *OsAPY2* demonstrated significant expression, and RNA-seq data revealed consistent outcomes (Fig 13), especially in cold conditions, its expression was very high. However, the real-time expression level was much more significant in response to cadmium and salinity stress. *OsAPY3* expression was substantially increased in heat and cold stress. Compared to the control condition, it was much greater during cold stress and demonstrated an 8-fold upregulation. Only heat stress raised the expression of *OsAPY4* (approximately eight-fold) and *7* (more than four-fold), whereas salinity and heat stress significantly downregulated the expression of *OsAPY9*. Under heat stress, RNA-seq data also revealed a higher level of *OsAPY4* expression. During cadmium, cold, salinity, and heat stress, *OsAPY6* expression was significantly enhanced, and RNA-seq data also exhibited upregulated expression under salinity and heat stress (Fig 13). Under cadmium, salinity, submergence, drought, and heat stress, *OsAPY8* demonstrated significant downregulation. Though the expression under cold conditions was higher, it was not significant. Its downregulated expression level during drought, heat, and submergence stress and the increased one in cold was also supported by RNA-seq data. According to RNA-seq data, prolonged exposure to submerged situations increased its expression. This result indicates a potential function for *OsAPY*s in regulating abiotic stress.

## Discussion

A noteworthy role is played by apyrase (*APY*) in regulating the growth of plants, developmental changes, and different stresses [18, 24]. The extracellular ATP (eATP) level rises when the plants are under any kind of stress which in turn increases ROS expression, triggers several other stress-induced genes, and can also cause apoptosis [5, 17]. Apyrase can hydrolyze eATP [137], and thus it has a major function in regulating eATP-mediated ROS production, and cellular apoptosis and in providing tolerance towards various stress conditions. Rice is the primary food consumed by the majority of people worldwide, and Asia alone contributes 90% to global rice production [138]. Different stresses are blamed for yield losses in commercially essential crops in many parts of the world. So, manipulating *APY*s can be a great step towards developing genetically modified multiple stress-tolerant rice and improving its annual production as *APY*s are associated with various developmental stages as well as stress responses of the plants. For this, the *APY* gene family should be studied intensively. Wheat, peanut and *Arabidopsis* have been extensively investigated for this gene family [2, 23, 31], but an investigation in rice is still not conducted. This study was designed to identify, characterize, and for analyzing the patterns of *APY* expression in rice.

Rice was found to have nine apyrase genes, as per this study, and all of these genes contained all the 5 ACRs [22]. An expanded insight into the significance of apyrase under abiotic and biotic stress was gained by the identification, characterization, and profiling of the expression of *APY* genes. These genes are different in their sequence length, molecular weight, pI value, and GRAVY value which means that this gene family is diverse. The GRAVY value of the proteins is negative, confirming the hydrophilic nature of the proteins [95].

Ecto-APYs are usually present on the plasma membrane and other cell surfaces, and endo-APYs are localized on ER, Golgi body, etc. [23, 139], and extracellular ATP is regulated by both endo- and ecto-APYs [19, 140]. In this investigation, the proteins were present on the plasma membrane, mitochondria, endoplasmic reticulum, and chloroplast, suggesting that both endo-APYs and ecto-APYs are present in rice [2, 23]. Nevertheless, the localization of OsAPY3, 4, and 9 varies according to the two different tools. Findings of CELLO [59] indicated there are three ecto-APYs (OsAPY2, 4, 5), but WoLF PSORT [58] suggested there are two ecto-APYs (OsAPY2, 5). It will take further research to find their precise position.

During evolution, gene duplication has a significant contribution to the process of gene expansion, and during plant development and growth, gene duplication can assist plants in adapting to various conditions [141]. The study of duplication events identified six duplication occurrences within the genome. The evolution of these genes occurred under the influence of purifying selection, which was suggested by the value of Ka/Ks and it was less than 1 [96]. These duplications occurred in genes situated on different chromosomes, indicating the segmental type of duplication being the main force of diversification [96]. Although segmental duplication preserves the primary functional group, it leads to differentiation in the manner of duplicated genes [142].

*OsAPY*s were related to *APY*s from *Arabidopsis*, peanut and wheat by constructing a phylogenetic tree in order to better understand their structure and functions. It was found that the phylogenetic tree was composed of three distinct groupings. According to earlier research on *Arabidopsis*, peanut and wheat *APY*s, this conclusion is in accordance [2, 23, 31]. None of the *OsAPY*s was categorized as members of any new group. *OsAPY* was found to be a very archaic family of genes, originating before even the splitting of monocotyledon (rice and wheat) and dicotyledon (*Arabidopsis*, peanut) plants because the *OsAPY* genes shared an equal number of groups with the *AtAPY* and *AhAPY* genes and with the *TaAPY* genes [143].

The number and orientation of exon-introns in plant genes play an essential role in evolution [144]. Gene structure analysis predicted that there was no intronless gene which indicates their high expression, and this gene family is an ancient one and not recently evolved [145, 146]. *OsAPY5* contained the lowest exon and intron, whereas *OsAPY6* contained the maximum exons and introns. Although *OsAPY1*, *3*, and *6* belonged to group I of the phylogenetic tree, their exon-intron structure was different from the other members of group I. *OsAPY2* and *OsAPY4* belonged to group II, but their gene structure was consistent with the other members of group I. *OsAPY5* which belonged to group III had a completely different exon-intron structure.

For proteins to function and to be particular, conserved motifs are necessary. Finding the structural patterns that are common to many proteins will help us better understand how proteins interact [147]. Conserved motif analysis revealed that motif 1, 5, 8, and 10 were conserved across all OsAPYs; motif 2 was present in all the OsAPYs except OsAPY2. Motif 3, 4, 6, 7, and 9 were the least distributed. These were only found in phylogenetic group I, and each group’s members had a remarkably comparable structure. Their function analysis revealed that 8 of the identified motifs are members of the GDA1-CD39 family, which confirms that the identified genes are members of this GDA1-CD39 nucleosidephosphatase superfamily.

The percentage of alpha-helix, beta-sheet, isolated beta bridge, turn, coil, and 310-helix was similar in all the proteins. The secondary structure analysis showed the dominance of alpha-helix over other structures, which implied the stability of the protein structure [135]. The membrane-spanning motif numbers varied from 0 to 2, which are similar to the previous findings [2, 23]. Proteins containing one motif had only N- terminal MSM. The proteins with two motifs had both N- and C- terminal MSMs except OsAPY6, which had only 2 N-terminal MSMs. N- as well as C- terminal MSMs are found in OsAPY2, 4, and 5. These proteins with both the N- and C- terminal MSMs were considered as ecto-APYs according to CELLO [59]. In the earlier studies on human APYs, proteins containing both the MSMs were also predicted to be ecto-APYs [2]. This finding is in accordance with the result obtained from CELLO [59] and TMHMM web servers [81].

The tertiary structure analysis showed that all the proteins’ projected structures were of high quality, which was validated by a number of tools. These structures could be utilized in analyzing the proteins more precisely in future research. Their docking analysis showed that there existed four categories of bonds between the proteins and their ligand ATP. The protein-ligand complex’s stability is supported by its high number of hydrogen bonds because these bonds normally aid in the stability of such complexes [148].

In the promoter region of a gene, cis-elements and transcription factors (TFs) interact, where they initiate gene transcription by assembling into the transcription initiation complex and then activating the RNA polymerase [104]. Sixty-nine cis-elements were identified in *OsAPY*s and categorized into eight groups. They played a crucial role in controlling different stresses and developmental phases, demonstrating the involvement of OsAPYs in these circumstances. A trait’s development and expression in plants are influenced by the gene’s chromosomal location [110]. Genes were unequally distributed throughout the genome in this study, and all of the OsAPY genes were found on chromosomes 3, 7, 8, 10, 11, and 12, with chromosome 11 having the most OsAPY genes.

We identified six distinct hormone-sensitive cis-elements in the promoter region, including those that are responsive to auxin, gibberellin, salicylic acid, ethylene, abscisic acid and methyl-jasmonate. The involvement of *APY* in maintaining the level of auxin and ethylene [149] and in controlling the closure of stomata in drought conditions induced by abscisic acid [150] was well documented. Additionally, there is ample evidence regarding the crosstalk between ethylene, gibberellin, and auxin [151–153]. These earlier results and the current investigation show that *OsAPY*s are probably important in the associated hormonal pathways.

The responsiveness of plants to external stimuli is tightly regulated by miRNA-mediated regulation of genes [39]. The OsAPY family members of rice were the targets of 103 miRNAs found in this investigation. Only 23 miRNAs specifically targeted more than one gene; the rest were gene-specific. osa-miR5819 targeted four different genes. It implies that osa-miR5819 could have an important function in the expression of *OsAPY*s. Maximum of the miRNAs were found to be downregulated in many stresses and tissues, with exceptions of osa-miR159a.1, osa-miR5532, osa-miR408-3p and osa-miR3979-3p. Comparatively, the miRNAs displayed decreased expression in response to a variety of stresses; this suggests that the miRNA-mediated regulation of the OsAPY genes may play a decisive role in how plants adapt to changing stress factors and growth conditions.

As per the 3K SNP search database, the prevalence of nonsynonymous SAPs in *OsAPY*s is significantly greater for indica rice cultivars than other rice cultivars. *OsAPY1*, *5*, and *8* showed no SAPs in the studied varieties. Results indicate that the *OsAPY1*, *5*, and *8* are substantially conserved among the studied genotypes. *OsAPY2* contained the maximum number of SAPs which was 5 SAPs. The studied varieties are stress-responsive [39, 77] the presence of SAPs indicates their stress responsiveness might be related to these SAPs [154].

OsAPY2 was found to have elevated expression levels under cold, heat, salt, and submergence stress, and the SNP analysis revealed that it contained 6 different SNPs that resulted in SAPs and 5 of them were cold, heat, salt, blight, pest, and blast fungus-resistant ones (Table 4). This finding strengthened the possibility of its involvement in those stresses. They were found to be involved in different types of stress resistance depending on the variety of rice. The varieties in which these SNPs appeared were resistant to the respective stresses indicating their possible role in those varieties for the specific stress. Though the expression profiling of OsAPY3 and 4 revealed their upregulated expression under heat stress, their SNPs did not show any involvement in heat stress. OsAPY9 did not exhibit any significant expression under salt and heat stress in RT-qPCR analysis, but the SNPs located in the gene were confirmed to be salt, heat, and cold-resistant ones for the specific rice varieties. This finding implies that these SNPs might play a role in creating resistance against those stress conditions. SNPs in OsAPY6 and 7 were mainly involved in creating resistance against the biotic stress conditions suggesting their involvement in providing resistance against these biotic stresses. The SNPs for OsAPY2 at 14938900, 14940235, 14940827, 14941519, and 14941823bp; for OsAPY3 at 28963907bp; for OsAPY6 at 1204513bp; for OsAPY7 at 1226429bp; for OsAPY9 at 1107655, 1109364, and 1109753bp were unique for a particular rice variety under a specific stress condition (Table 4). Due to this specificity, they can act as markers for screening the related rice genotype under specific stress conditions.

The involvement of *OsAPY*s in various tissues during the development and growth of rice was determined by an analysis of their expression patterns (Fig 11) using RNA-seq data. *OsAPY1-4*, *9* showed higher levels of gene expression in inflorescence, implying their possible function in flower development. Previous studies have demonstrated the function of APY in flowers [25, 155]. *OsAPY2* and *4* had an abundant expression in anther, whereas *OsAPY1*, *3–5* showed elevated expression in the pistil, suggesting their potential roles in the germination of pollen, fertilization, and reproduction, respectively. The activity of *APY*s in pollen and pistils was reported in *Arabidopsis* [25, 33]. *OsAPY9* expression was found to be higher in the leaves and seedling stage, indicating that it plays a potential function in leaf development and early phases of plant growth. The involvement of *APY*s in leaves has previously been documented in *Medicago truncatula* [155] and *Arabidopsis* [25]. Another research on pea seedlings found that *APY*s were involved in early growth and development [156].

To further consider the dynamics of the *OsAPY*s in various biotic and abiotic stresses, their expression profiles via RNA-seq data were studied. The activity of *OsAPY*s was investigated in response to bacteria, fungus, and viruses, and *OsAPY1-5*, and *8* exhibited higher activation. *APY*s have been discovered to function in defensive and symbiotic relationships between plants and microorganisms [157]. *OsAPY1-5* demonstrated higher activity under blight-causing bacteria *Xanthomonas oryzae* pv. *oryzicola*. Both types of viral infections elevated the expression of *OsAPY2*, *3* and *5*; under fungal infection, the expression of *OsAPY2*, *5*, and *8* increased. This discovery is in line with an earlier study that found the pea *APY* gene (*PsAPY1*) to be engaged in delivering protection against fungal infections [158]. The analysis of cis-elements showed that in the promoter region of *OsAPY*s, WRE3, WUN motifs, and W-box, which regulate biotic stresses in plants were present. The presence of such motifs corroborates our findings regarding the involvement of these genes in providing a defense under such stress conditions.

To identify the role of *OsAPY*s in abiotic stresses, the data retrieved from the RNA-seq database and the RT-qPCR analysis result were correlated. *OsAPY2* and *8* were essential genes concerning cold stress in both cases, suggesting their potential role under cold condition. In cold condition, *APY* involvement has been reported in *Populus euphratica* (*PeAPY2*) [137]. *OsAPY1*, *2*, and *5* showed increased activity under submergence stress suggesting their probable involvement in this condition. *OsAPY1-6* had upregulated expression under heat stress, suggesting these genes’ involvement in heat stress. Under salt stress, *OsAPY2* and *6* demonstrated elevated expression in both cases, indicating their role in helping the plant survive under salt stress. Similar results were found in wheat, where majority of the *APY* genes had higher expression after being exposed to salt for 12 hours in both roots and leaves [23]. *OsAPY5* showed higher expression under drought conditions indicating its role in helping plants cope with this condition, as the role of *APY* was previously reported in a study where the pea ectoapyrase was expressed in soybean and *Arabidopsis* and it resulted in better growth and tolerance towards drought conditions [159]. Presence of many abiotic stress-responsive cis-elements supports the notion concerning the role of *APY*s in abiotic stress conditions. Gene expression is one of the complicated biological processes, and it requires further research for elucidating the mechanisms of the *OsAPY*s behind the regulation of several stresses. These findings indicate the function of *OsAPY*s under diverse biotic and abiotic stresses, and so this gene family could prove to be an important candidate for genetic engineering that can provide protection against various stress conditions.

## Conclusion

In this study, the rice *APY* gene family has been identified, characterized, and its expression profiling has been done extensively. Nine genes were identified, and they were found to be located on chromosomes 3, 7, 8, 10, 11, and 12. Phylogenetic analysis grouped the nine identified *OsAPY* genes into three groups, and the identified cis-elements revealed their involvement in different stress conditions, hormonal regulation, and developmental stages. This ancient gene family evolved via segmental duplication and some SAPs were present in the stress-responsive varieties, some of which might act as markers for screening a certain rice genotype under specific stresses. Four different categories of bonds were identified between the OsAPYs and ATP, suggesting strong docking interactions between them. miRNA analysis helped in understanding the function of miRNA in modulating *OsAPY* activity, while expression profiling via RNA-seq data unveiled the role of *OsAPY*s under various growth phases as well as stresses. The expression analysis using RT-qPCR data also confirmed the response of *OsAPY*s in various abiotic stress conditions, especially in heat stress. *OsAPY2* and *5* demonstrated increased expression when RNA-seq and RT-qPCR data were compared, suggesting they could be potential candidates for further study. These findings would give the insight to broaden the understanding and knowledge regarding *APY*s in rice as well as provide a foundation in future research regarding *OsAPY*s and also in the genomic alterations towards the improvement of rice and eventually developing stress-tolerant varieties.

## Supporting information

S1 Fig Apyrase conserved region (ACR) analysis of the OsAPYs. The alignment was done using MEGA X and visualized via GeneDoc. The navy blue, pink, and aqua colors indicate the amino acids that are conserved 100%, 80%, and 60%, respectively, and the red-colored boxes depict the apyrase conserved region (ACR).
(TIF)

S2 Fig Ramachandran plot of the tertiary structures of OsAPYs. The Ramachandran plot was generated via PROCHECK. Residues in the most favored, additional allowed, generously allowed, and disallowed regions are specified via red, yellow, pale yellow, and white colors, respectively.
(TIF)

S3 Fig ERRAT plot of the tertiary structures of OsAPYs. ERRAT plot was generated via ERRAT. Yellow bars indicate the segment of the proteins which could be excluded at a 95% confidence level, and the red bars denote the ones at a 99% confidence level. The section with a lower error rate is marked by white bars.
(TIF)

S4 Fig Z-score plot of the tertiary structures of OsAPYs. The Z-score plot was generated via ProSA-web. The light blue color indicates the Z-score of the proteins measured by X-ray crystallography, and the dark blue color indicates the Z-score of the proteins measured by nuclear magnetic resonance (NMR) spectroscopy. Black dots indicate the Z-score of each protein.
(TIF)

S5 Fig Energy plot of the tertiary structures of OsAPYs. The energy plot was generated via ProSA-web. The dark green line represents the energy averaged across each fragment of 40 residues, and the light green line depicts the one across each fragment of 10 residues.
(TIF)

S6 Fig Ramachandran plot of the protein (OsAPYs)- ligand (ATP) complex structure. The Ramachandran plot was generated via PROCHECK. Residues in the most favored, additional allowed, generously allowed, and disallowed regions are specified via red, yellow, pale yellow, and white colors, respectively.
(TIF)

S7 Fig ERRAT plot of the protein (OsAPYs)- ligand (ATP) complex structure. ERRAT plot was generated via ERRAT. Yellow bars indicate the segment of the proteins which could be excluded at a 95% confidence level, and the red bars denote the ones at a 99% confidence level. The section with a lower error rate is marked by white bars.
(TIF)

S1 Table Description of the conserved motifs in OsAPYs. (DOCX)

S2 Table Duplicated *APY* genes and the probable dates of duplication blocks in rice. (DOCX)

S3 Table The location of all the *OsAPY*s in different chromosome, their position, and orientation. (DOCX)

S4 Table List of miRNAs targeting different *OsAPY*s and their mode of inhibition. (DOCX)

S5 Table The percentage of alpha-helix, beta-sheet, beta bridge, turn, coil, 310 helix, and the position of the transmembrane motif in the OsAPY members. (DOCX)

S6 Table Validation score of OsAPY tertiary structure via different tools. (DOCX)

S7 Table Docking score of the docked complexes generated via HDOCK server. (DOCX)

S8 Table Number of bonds formed between OsAPYs and ATP according to PDBSum analysis. (DOCX)

S9 Table Analysis of the interaction of OsAPY proteins with their ligand (ATP). (DOCX)

S10 Table List of primers used in RT-qPCR analysis. (DOCX)
