# Supplementary figures and images for: Identification, characterization of Apyrase (APY) gene family in rice (Oryza sativa) and analysis of the expression pattern under various stress conditions
Source: PLoS One. 2023 May 10;18(5):e0273592. doi: 10.1371/journal.pone.0273592 (PMC10171694; doi:10.1371/journal.pone.0273592)

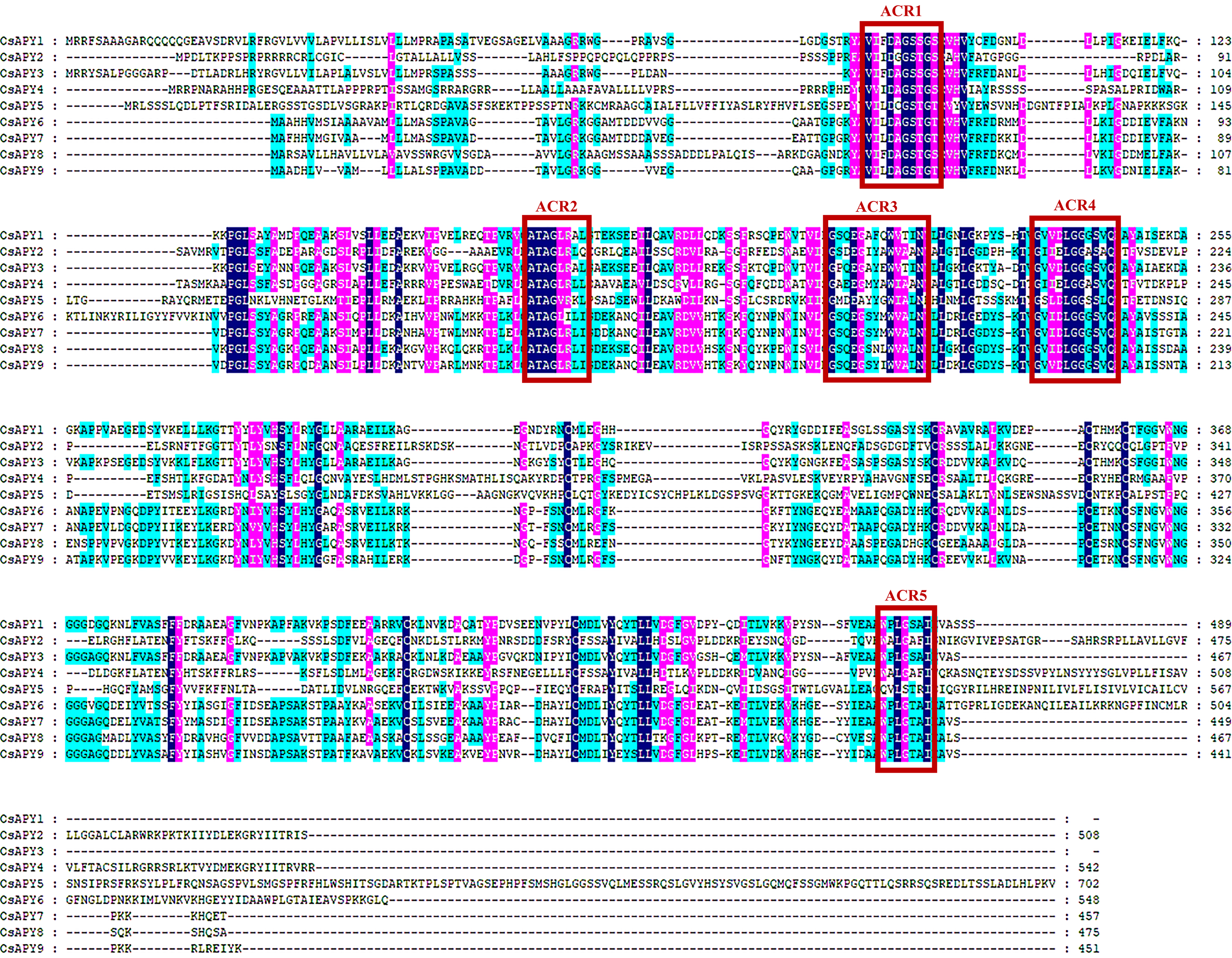

Supplement: S1 Fig — The alignment was done using MEGA X and visualized via GeneDoc. The navy blue, pink, and aqua colors indicate the amino acids that are conserved 100%, 80%, and 60%, respectively, and the red-colored boxes depict the apyrase conserved region (ACR). (TIF) [file pone.0273592.s001.tif]

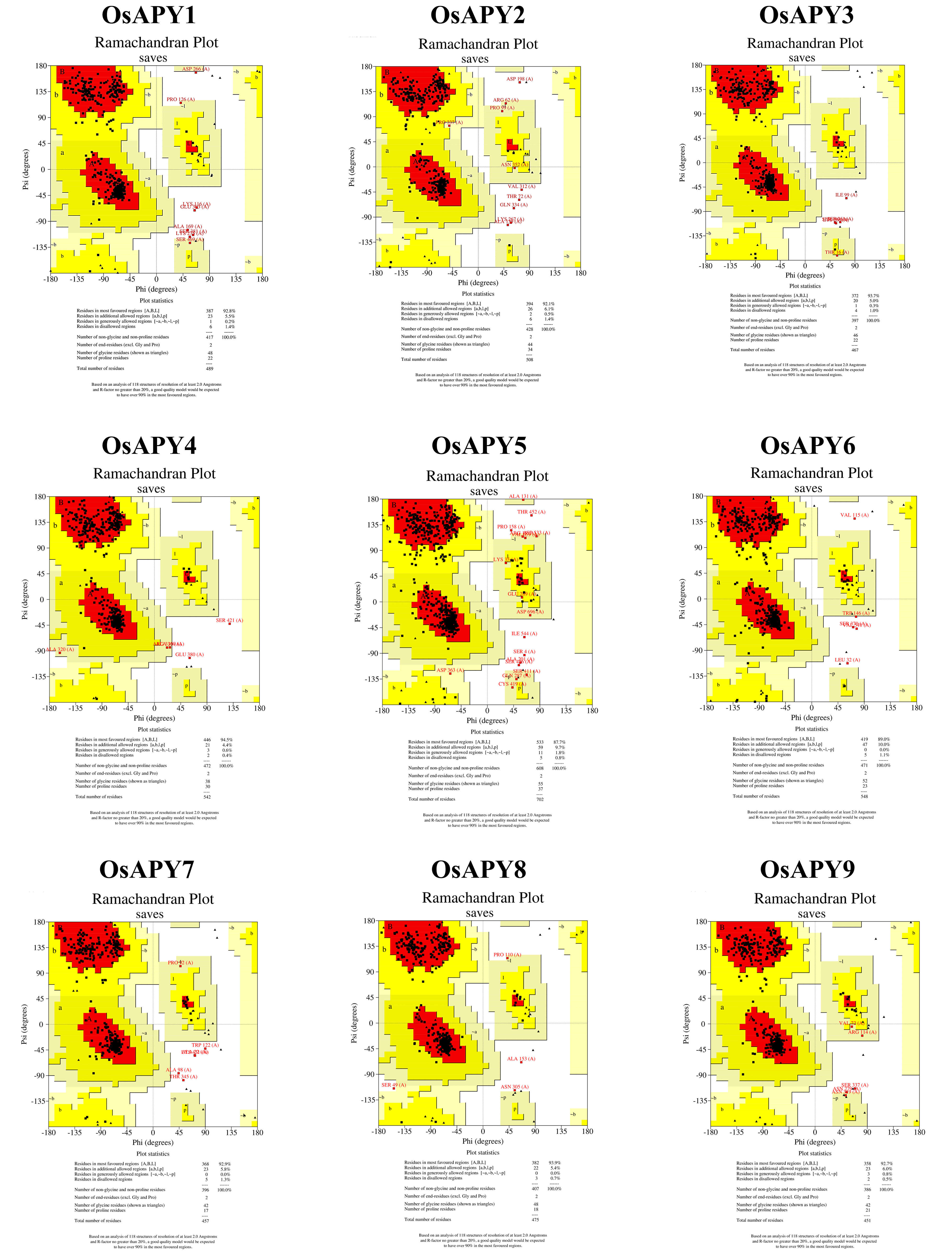

Supplement: S2 Fig — The Ramachandran plot was generated via PROCHECK. Residues in the most favored, additional allowed, generously allowed, and disallowed regions are specified via red, yellow, pale yellow, and white colors, respectively. (TIF) [file pone.0273592.s002.tif]

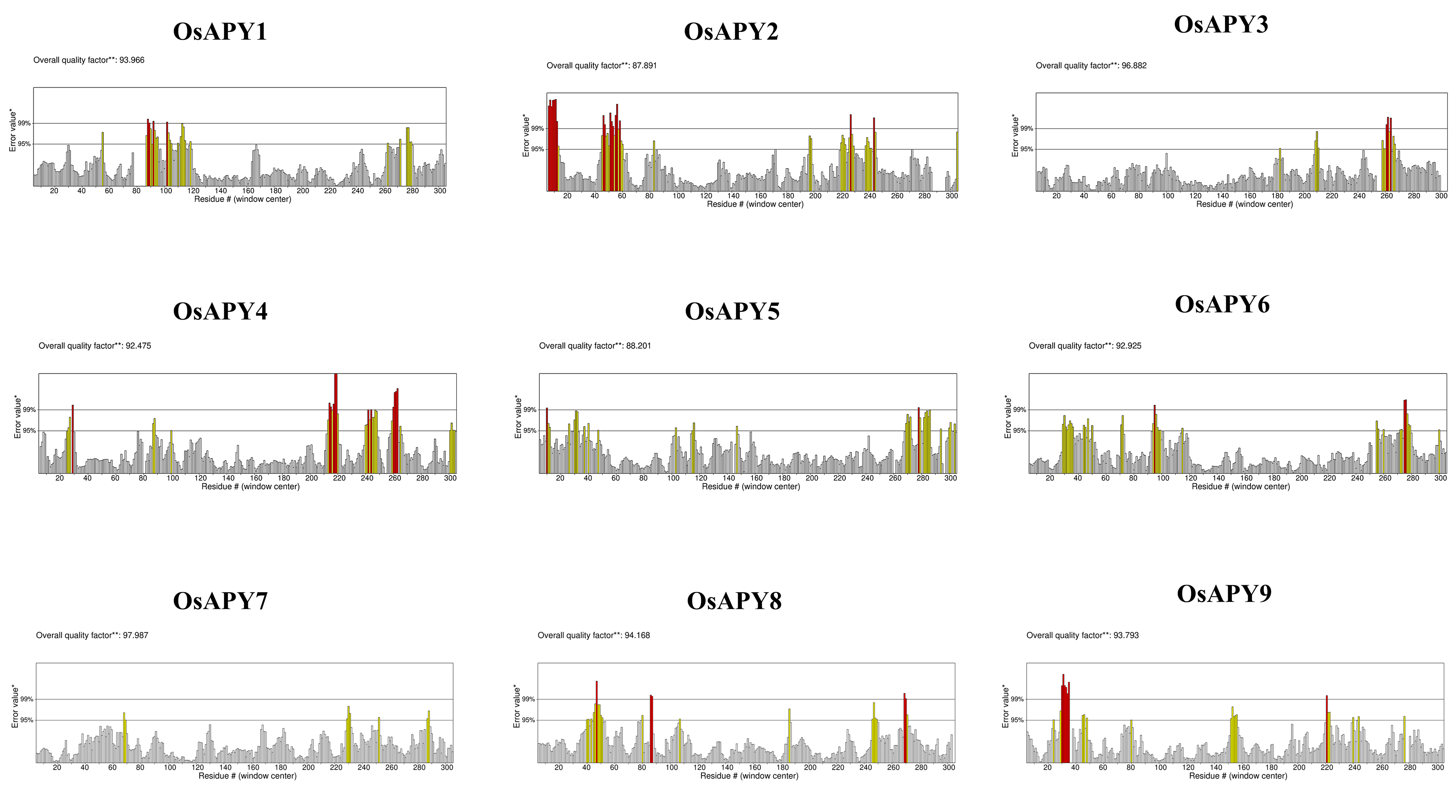

Supplement: S3 Fig — ERRAT plot was generated via ERRAT. Yellow bars indicate the segment of the proteins which could be excluded at a 95% confidence level, and the red bars denote the ones at a 99% confidence level. The section with a lower error rate is marked by white bars. (TIF) [file pone.0273592.s003.tif]

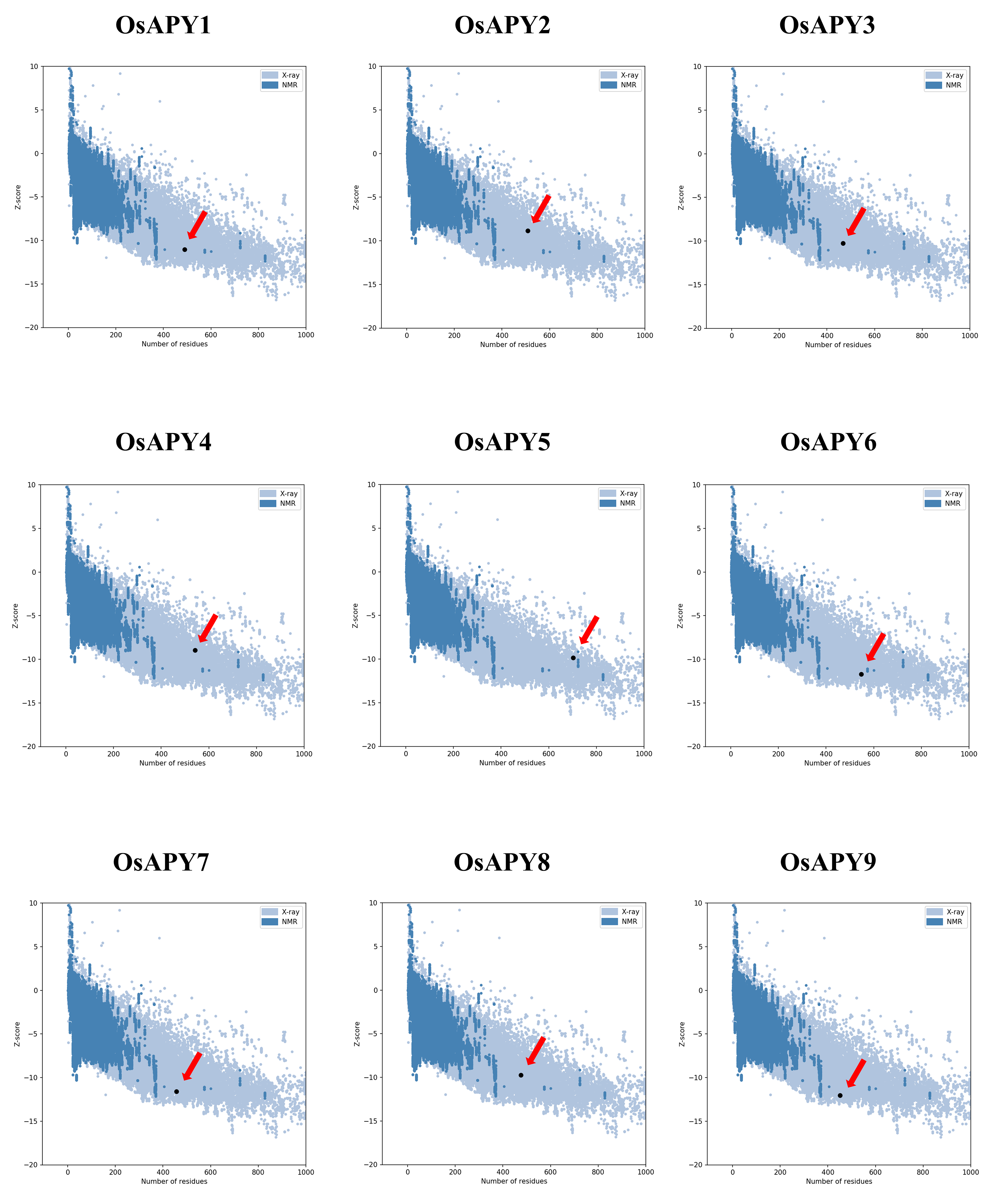

Supplement: S4 Fig — The Z-score plot was generated via ProSA-web. The light blue color indicates the Z-score of the proteins measured by X-ray crystallography, and the dark blue color indicates the Z-score of the proteins measured by nuclear magnetic resonance (NMR) spectroscopy. Black dots indicate the Z-score of each protein. (TIF) [file pone.0273592.s004.tif]

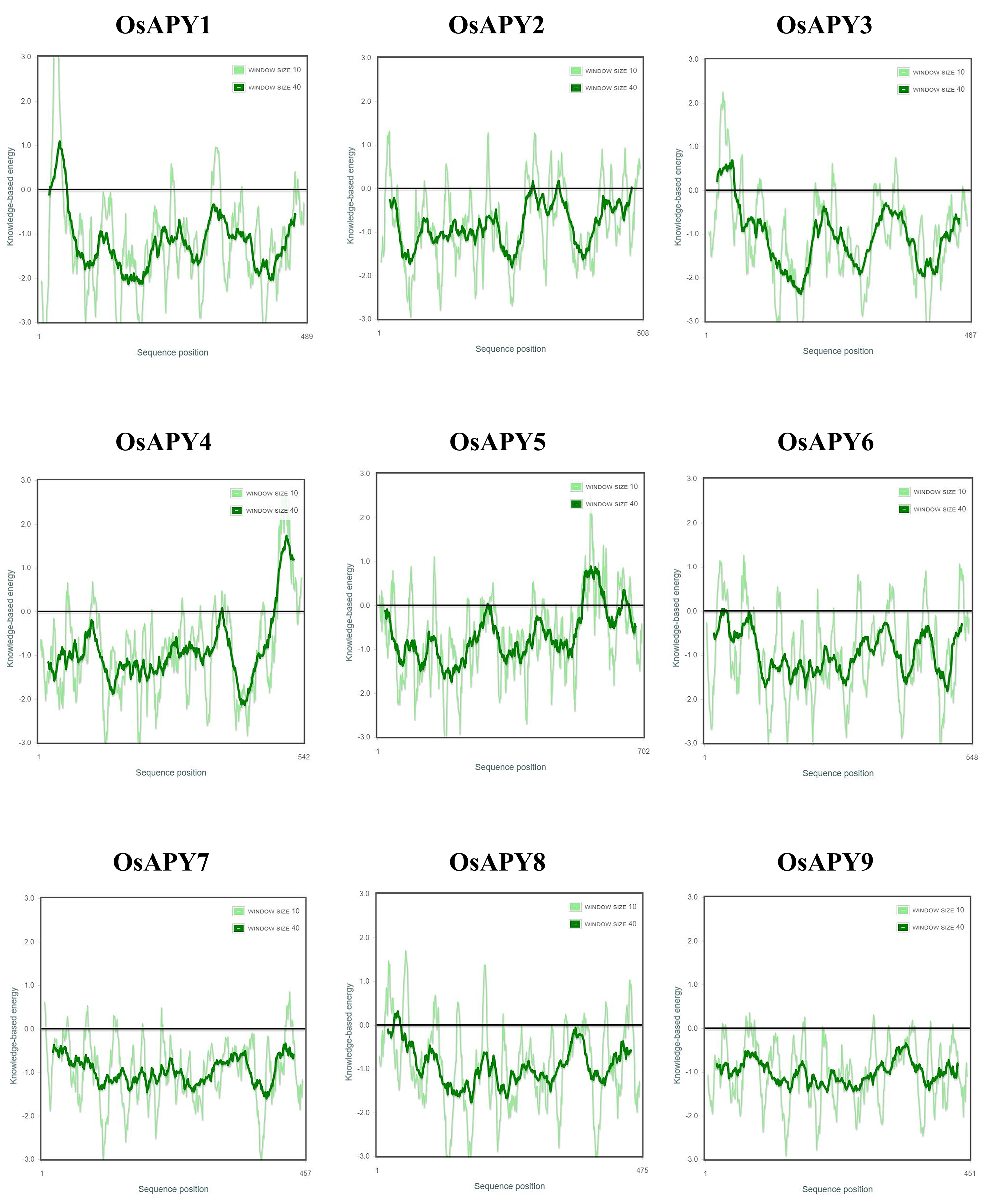

Supplement: S5 Fig — The energy plot was generated via ProSA-web. The dark green line represents the energy averaged across each fragment of 40 residues, and the light green line depicts the one across each fragment of 10 residues. (TIF) [file pone.0273592.s005.tif]

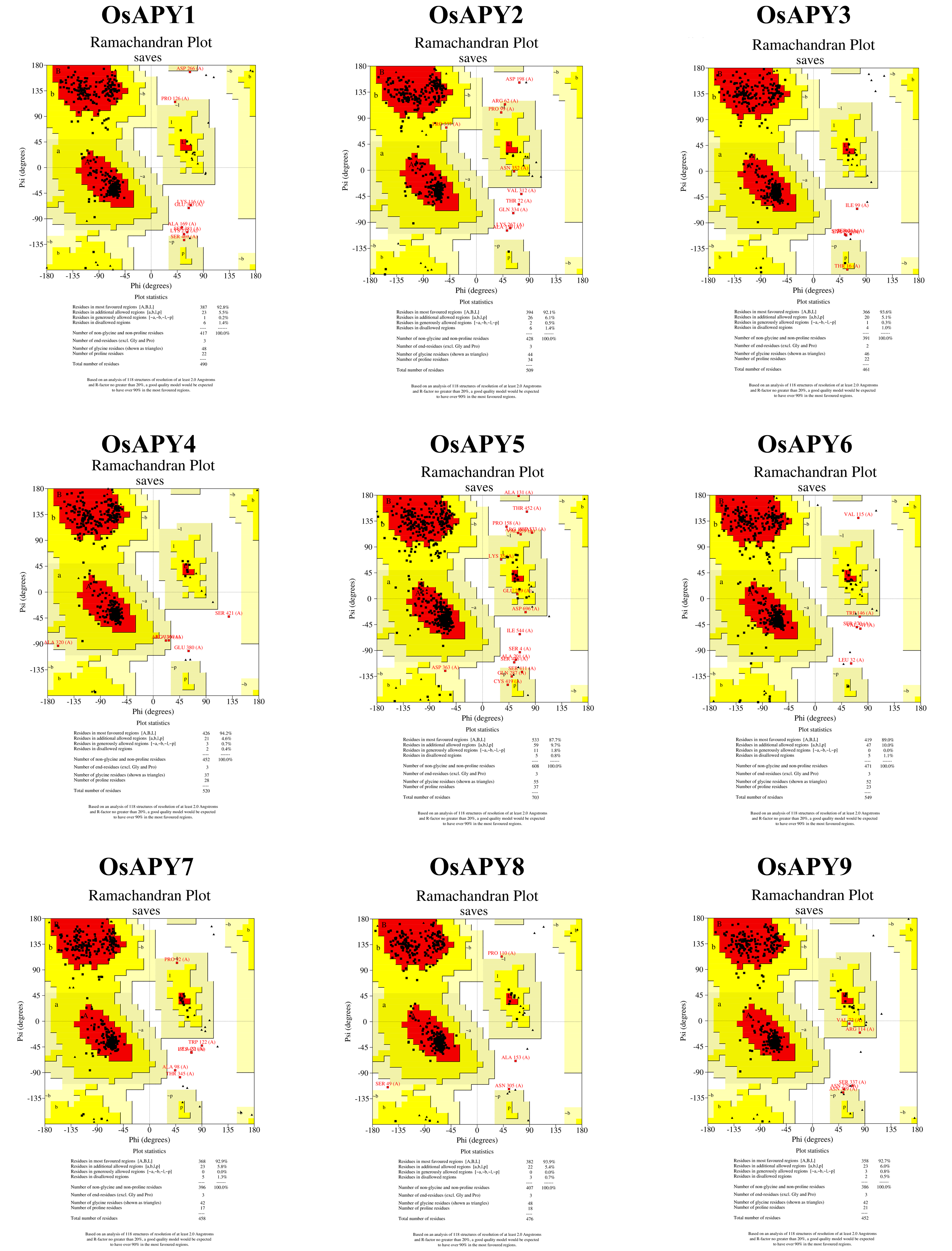

Supplement: S6 Fig — The Ramachandran plot was generated via PROCHECK. Residues in the most favored, additional allowed, generously allowed, and disallowed regions are specified via red, yellow, pale yellow, and white colors, respectively. (TIF) [file pone.0273592.s006.tif]

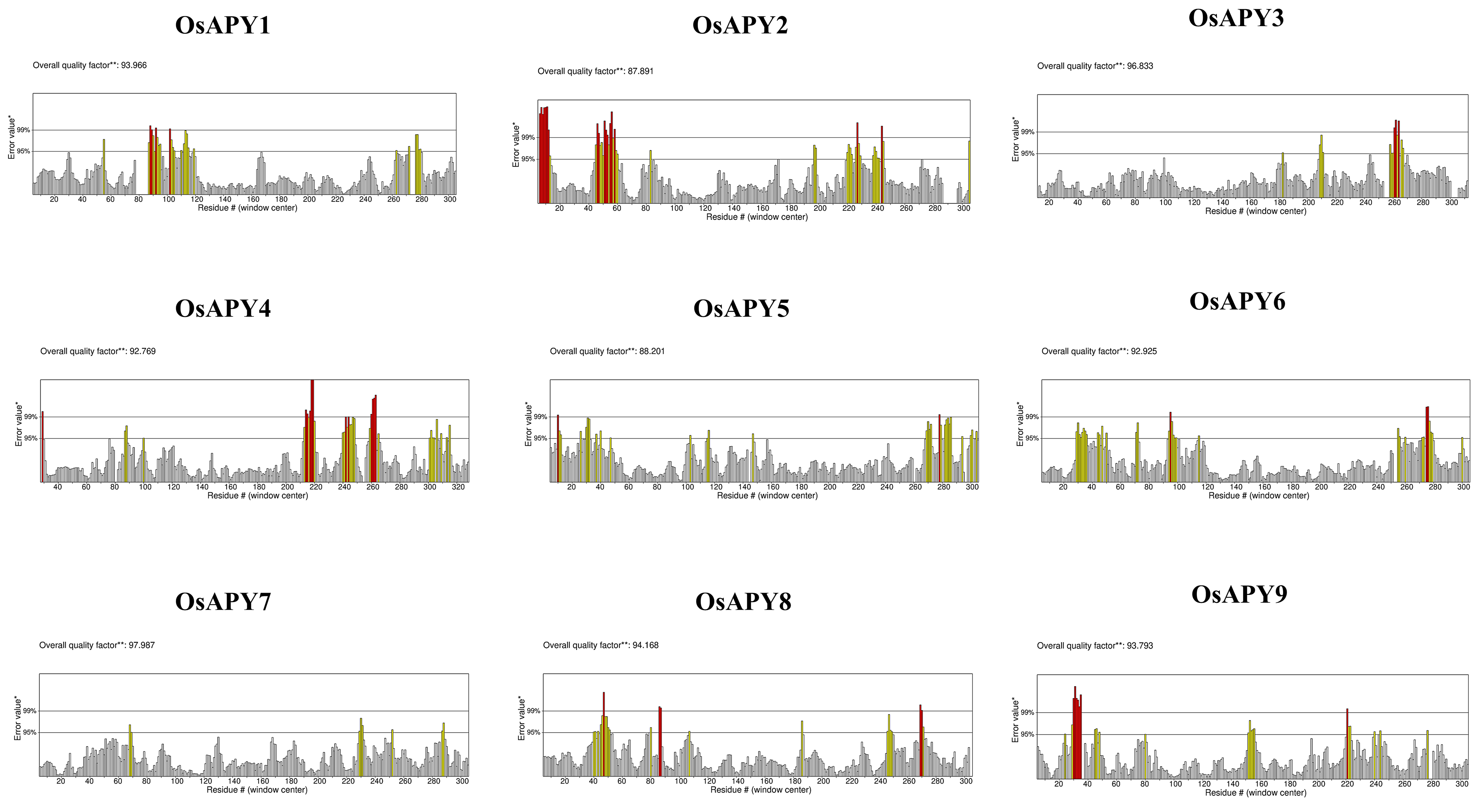

Supplement: S7 Fig — ERRAT plot was generated via ERRAT. Yellow bars indicate the segment of the proteins which could be excluded at a 95% confidence level, and the red bars denote the ones at a 99% confidence level. The section with a lower error rate is marked by white bars. (TIF) [file pone.0273592.s007.tif]
