# Supplementary material for: Identification, characterization of Apyrase (APY) gene family in rice (Oryza sativa) and analysis of the expression pattern under various stress conditions
Source: PLoS One. 2023 May 10;18(5):e0273592. doi: 10.1371/journal.pone.0273592 (PMC10171694; doi:10.1371/journal.pone.0273592)
Supplement: S1 Table — (DOCX) [file pone.0273592.s008.docx]

| **Motif** | **Sequence** | **Width** | **Description** |
| --- | --- | --- | --- |
| 1 | FQYQPDWITVLSGSQEGSYAWVALNYLLGKLGGDYSK | 37 | GDA1/CD39 (nucleoside phosphatase) family |
| 2 | PGLSSYAGRPQEAAKSJVPLLEKAKKVVPVELMKKTPLKLGATAGLRLJG | 50 | GDA1/CD39 (nucleoside phosphatase) family |
| 3 | GEDPYVTKEYLKGKDYNJYVHSYLHYGLLASRVEILKRKNG | 41 | GDA1/CD39 (nucleoside phosphatase) family |
| 4 | YLCMDLVYQYTLLVDGFGLEPTKEITLVEKVKHGEYYIEAAWPLGTAIEA | 50 | GDA1/CD39 (nucleoside phosphatase) family |
| 5 | GPGRYAVILDAGSTGSRVHVFRFDKNLDL | 29 | GDA1/CD39 (nucleoside phosphatase) family |
| 6 | CSFNGVWNGGGGAGQDDLYVASYFYDRASEAGFIBSEAPSAKSTPAAFK | 49 | GDA1/CD39 (nucleoside phosphatase) family |
| 7 | FSNCMLRGFSGKYKYNGEQYDASAAPQGADYHKCREDVVKALKLDAPCE | 49 | GDA1/CD39 (nucleoside phosphatase) family |
| 8 | TVGVIDLGGGSVQMAYAISEKLAAEAPKV | 29 | GDA1/CD39 (nucleoside phosphatase) family |
