# Supplementary material for: Identification, characterization of Apyrase (APY) gene family in rice (Oryza sativa) and analysis of the expression pattern under various stress conditions
Source: PLoS One. 2023 May 10;18(5):e0273592. doi: 10.1371/journal.pone.0273592 (PMC10171694; doi:10.1371/journal.pone.0273592)
Supplement: S2 Table — (DOCX) [file pone.0273592.s009.docx]

| **Duplicated Gene 1** | **Duplicated Gene 2** | **Ka** | **Ks** | **Ka/Ks** | **Duplication time (MYA)** | **Purifying Selection** | **Duplication type** |
| --- | --- | --- | --- | --- | --- | --- | --- |
| *OsAPY1* | *OsAPY9* | 0.4225 | 1.7895 | 0.2361 | 59.65 | Yes | Segmental |
| *OsAPY1* | *OsAPY3* | 0.1269 | 0.8597 | 0.1476 | 28.65 | Yes | Segmental |
| *OsAPY3* | *OsAPY9* | 0.4367 | 1.9982 | 0.2185 | 66.6 | Yes | Segmental |
| *OsAPY1* | LOC_Os11g03230 | 0.438 | 1.7011 | 0.2575 | 56.7 | Yes | Segmental |
| *OsAPY3* | LOC_Os11g03230 | 0.456 | 1.7176 | 0.2655 | 57.25 | Yes | Segmental |
| *OsAPY9* | LOC_Os11g03230 | 0.0258 | 0.0464 | 0.5560 | 1.54 | Yes | Segmental |
