# Supplementary material for: Identification, characterization of Apyrase (APY) gene family in rice (Oryza sativa) and analysis of the expression pattern under various stress conditions
Source: PLoS One. 2023 May 10;18(5):e0273592. doi: 10.1371/journal.pone.0273592 (PMC10171694; doi:10.1371/journal.pone.0273592)
Supplement: S3 Table — (DOCX) [file pone.0273592.s010.docx]

| **Gene Name** | **Chromosome Number** | **Chromosomal Location** | **Chromosomal Orientation** |
| --- | --- | --- | --- |
| *OsAPY1* | 3 | 12021782-12027329 | Reverse |
| *OsAPY2* | 3 | 14938457-14942303 | Forward |
| *OsAPY3* | 7 | 28963027-28967286 | Forward |
| *OsAPY4* | 8 | 21190207-21194423 | Forward |
| *OsAPY5* | 10 | 10637369-10641992 | Reverse |
| *OsAPY6* | 11 | 1202740-1208798 | Forward |
| *OsAPY7* | 11 | 1222800-1226701 | Forward |
| *OsAPY8* | 11 | 14390248-14398123 | Reverse |
| *OsAPY9* | 12 | 1107401-1110538 | Forward |
