# Supplementary material for: Identification, characterization of Apyrase (APY) gene family in rice (Oryza sativa) and analysis of the expression pattern under various stress conditions
Source: PLoS One. 2023 May 10;18(5):e0273592. doi: 10.1371/journal.pone.0273592 (PMC10171694; doi:10.1371/journal.pone.0273592)
Supplement: S4 Table — (DOCX) [file pone.0273592.s011.docx]

| **Gene Name** | **Number of targeting miRNA** | **miRNA** | **miRNA sequence** | **Length (nt)** | **Inhibition** |
| --- | --- | --- | --- | --- | --- |
| *OsAPY1* | 15 | osa-miR6249a | CGUGAAGAGCUCGCCGGCGGC | 21 | Cleavage |
|  |  | osa-miR6249b | CGUGAAGAGCUCGCCGGCGGC | 21 | Cleavage |
|  |  | osa-miR5082 | UGCGAUGAUGGCCGCGCGGGUUCA | 24 | Cleavage |
|  |  | osa-miR5337b | CUAGAACGGCAAGCAAUUUGA | 21 | Translation |
|  |  | osa-miR5508 | UAGAUGGCUGAUCUGGUGUGG | 21 | Cleavage |
|  |  | osa-miR1426 | AGAAUCUUGAUGAUGAUUAAA | 21 | Cleavage |
|  |  | osa-miR1437b-3p | GUGCUGGCGAGCUCCGGUGCCGCA | 24 | Translation |
|  |  | osa-miR167d-3p | GAUCAUGCUGUGCAGUUUCAUC | 22 | Cleavage |
|  |  | osa-miR1857-5p | UGGUUUUUUUGGAGCAUGAGG | 21 | Cleavage |
|  |  | osa-miR1862a | ACGAGGUUGGUUUAUUUUGGGACG | 24 | Cleavage |
|  |  | osa-miR1862b | ACGAGGUUGGUUUAUUUUGGGACG | 24 | Cleavage |
|  |  | osa-miR1862c | ACGAGGUUGGUUUAUUUUGGGACG | 24 | Cleavage |
|  |  | osa-miR1871 | AUGGCUCUGAUAUCAUGUUGGUUU | 24 | Cleavage |
|  |  | osa-miR5493 | AGCCGGGCUCGGUCGCGCGUG | 21 | Cleavage |
|  |  | osa-miR5499 | GAAGGAAGAAUCGUUAUGGAA | 21 | Cleavage |
| *OsAPY2* | 12 | osa-miR530-3p | AGGUGCAGAGGCAGAUGCAAC | 21 | Cleavage |
|  |  | osa-miR2098-3p | CGGUUUGUCAAGCGGAGUGC | 20 | Cleavage |
|  |  | osa-miR5082 | UGCGAUGAUGGCCGCGCGGGUUCA | 24 | Cleavage |
|  |  | osa-miR529b | AGAAGAGAGAGAGUACAGCUU | 21 | Cleavage |
|  |  | osa-miR5532 | AUGGAAUAUAUGACAAAGGUGG | 22 | Cleavage |
|  |  | osa-miR1858a | GAGAGGAGGACGGAGUGGGGC | 21 | Translation |
|  |  | osa-miR1858b | GAGAGGAGGACGGAGUGGGGC | 21 | Translation |
|  |  | osa-miR1870-3p | UUUAGGGCUAAUUCAGCAUGAACA | 24 | Translation |
|  |  | osa-miR2097-5p | AGAGAUGGGACGGGCAGGGAAG | 22 | Cleavage |
|  |  | osa-miR444d.3 | UUGUGGCUUUCUUGCAAGUUG | 21 | Cleavage |
|  |  | osa-miR5159 | AACUAGAGUGGGUCAACGGGUACC | 24 | Cleavage |
|  |  | osa-miR5819 | AGGACGAGGGGAACGGCGGCG | 21 | Cleavage |
| *OsAPY3* | 6 | osa-miR5819 | AGGACGAGGGGAACGGCGGCG | 21 | Cleavage |
|  |  | osa-miR1425-3p | CAGCAAGAACUGGAUCUUAAU | 21 | Cleavage |
|  |  | osa-miR1858a | GAGAGGAGGACGGAGUGGGGC | 21 | Cleavage |
|  |  | osa-miR1858b | GAGAGGAGGACGGAGUGGGGC | 21 | Cleavage |
|  |  | osa-miR2096-5p | UGCCGAUUUCCCCCUCGGGCG | 21 | Cleavage |
|  |  | osa-miR5514 | UCCCAGAGCUUUGGCCGUCGC | 21 | Cleavage |
| *OsAPY4* | 15 | osa-miR169r-3p | UGGCAAGUCUCCUCGGCUACC | 21 | Cleavage |
|  |  | osa-miR2925 | UGGCGGCCGCGGGCUUCGU | 19 | Cleavage |
|  |  | osa-miR1846a-5p | AGUGAGGAGGCCGGGGCCGCU | 21 | Cleavage |
|  |  | osa-miR1846b-5p | AGUGAGGAGGCCGGGGCCGCU | 21 | Cleavage |
|  |  | osa-miR1846c-5p | AGUGAGGAGGCCGGGGCCGCU | 21 | Cleavage |
|  |  | osa-miR159f | CUUGGAUUGAAGGGAGCUCUA | 21 | Translation |
|  |  | osa-miR2873b | UUGGACUUGAGAUUUGGUAUG | 21 | Cleavage |
|  |  | osa-miR159a.1 | UUUGGAUUGAAGGGAGCUCUG | 21 | Translation |
|  |  | osa-miR159b | UUUGGAUUGAAGGGAGCUCUG | 21 | Translation |
|  |  | osa-miR2091-3p | CAUACAUUGCCUCCUAGGCUUG | 22 | Translation |
|  |  | osa-miR2098-3p | CGGUUUGUCAAGCGGAGUGC | 20 | Translation |
|  |  | osa-miR2870 | UAAUCAGUUUGGGGAGACAAA | 21 | Cleavage |
|  |  | osa-miR2927 | UGUCGUCGUCGAUGGAGCCCAUG | 23 | Cleavage |
|  |  | osa-miR3979-3p | CUUCGGGGGAGGAGAGAAGC | 20 | Translation |
|  |  | osa-miR812o-5p | CGUGUUCAACGUUUGACUGUC | 21 | Translation |
| *OsAPY5* | 23 | osa-miR2099-5p | UGAAUAUGUUUGUACAAGCUUU | 22 | Cleavage |
|  |  | osa-miR5075 | UUCUCCGUCGCCGCCGUCCGC | 21 | Cleavage |
|  |  | osa-miR169r-5p | UAGCCAAGGAUGAUUUGCCUG | 21 | Cleavage |
|  |  | osa-miR1855 | AGCACUGGAGUAGCCAAGAGA | 21 | Cleavage |
|  |  | osa-miR408-3p | CUGCACUGCCUCUUCCCUGGC | 21 | Cleavage |
|  |  | osa-miR5152-5p | GUAGGGAUAGGCAUGAUCUCU | 21 | Cleavage |
|  |  | osa-miR5490 | UUGGAUUUUUAUUUAGGACGG | 21 | Translation |
|  |  | osa-miR5532 | AUGGAAUAUAUGACAAAGGUGG | 22 | Cleavage |
|  |  | osa-miR5815 | AAUGUUAUGGACACUAGAUGACAU | 24 | Cleavage |
|  |  | osa-miR166b-5p | GGAAUGUUGUCUGGCUCGGGG | 21 | Translation |
|  |  | osa-miR169i-5p.2 | UGGUGAUAAGGGUGUAGCUCUG | 22 | Cleavage |
|  |  | osa-miR171f-5p | UGUUGGCAUGGUUCAAUCAAA | 21 | Cleavage |
|  |  | osa-miR1846a-5p | AGUGAGGAGGCCGGGGCCGCU | 21 | Cleavage |
|  |  | osa-miR1846b-5p | AGUGAGGAGGCCGGGGCCGCU | 21 | Cleavage |
|  |  | osa-miR1846c-5p | AGUGAGGAGGCCGGGGCCGCU | 21 | Cleavage |
|  |  | osa-miR1877 | AGAUGACAUGUGAAUGAUGAGGGG | 24 | Translation |
|  |  | osa-miR2101-3p | AUUUAACUCAAGUGAGCAUUGU | 22 | Cleavage |
|  |  | osa-miR393b-3p | UCAGUGCAAUCCCUUUGGAAU | 21 | Cleavage |
|  |  | osa-miR415 | AACAGAACAGAAGCAGAGCAG | 21 | Translation |
|  |  | osa-miR5149 | GAGGAGCUGUGACGAUUUGGGA | 22 | Cleavage |
|  |  | osa-miR5541 | UCAAGUGGUGUACUCUAAAGA | 21 | Cleavage |
|  |  | osa-miR5821 | UGGACGGAGCGAUGGUGGGCG | 21 | Cleavage |
|  |  | osa-miR7695-3p | ACGUGAUGUGCCACGUAGGCA | 21 | Translation |
| *OsAPY6* | 13 | osa-miR5340 | UGAUGACGUGGAUGAAUUUCAAA | 23 | Cleavage |
|  |  | osa-miR171a | UGAUUGAGCCGCGCCAAUAUC | 21 | Cleavage |
|  |  | osa-miR171i-3p | GGAUUGAGCCGCGUCAAUAUC | 21 | Cleavage |
|  |  | osa-miR1847.2 | UGGCCCACAUGUUAGUGCCACAAC | 24 | Cleavage |
|  |  | osa-miR3980a-3p | CUGGCCGAGGCCGUCGAUUCU | 21 | Cleavage |
|  |  | osa-miR3980b-3p | CUGGCCGAGGCCGUCGAUUCU | 21 | Cleavage |
|  |  | osa-miR444f | UGCAGUUGUUGCCUCAAGCUU | 21 | Cleavage |
|  |  | osa-miR444f | UGCAGUUGUUGCCUCAAGCUU | 21 | Cleavage |
|  |  | osa-miR5338 | UGAAGCUUCAGUUGGUUGUAU | 21 | Cleavage |
|  |  | osa-miR5543 | UAUGAAUGGUAUAUUUUCUUG | 21 | Translation |
|  |  | osa-miR821a | AAGUCAUCAACAAAAAAGUUGAAU | 24 | Cleavage |
|  |  | osa-miR821b | AAGUCAUCAACAAAAAAGUUGAAU | 24 | Cleavage |
|  |  | osa-miR821c | AAGUCAUCAACAAAAAAGUUGAAU | 24 | Cleavage |
| *OsAPY7* | 8 | osa-miR5338 | UGAAGCUUCAGUUGGUUGUAU | 21 | Cleavage |
|  |  | osa-miR5511 | CAUAUCCCAGCUGUUUCGGCC | 21 | Cleavage |
|  |  | osa-miR1847.2 | UGGCCCACAUGUUAGUGCCACAAC | 24 | Cleavage |
|  |  | osa-miR444f | UGCAGUUGUUGCCUCAAGCUU | 21 | Cleavage |
|  |  | osa-miR5152-5p | GUAGGGAUAGGCAUGAUCUCU | 21 | Cleavage |
|  |  | osa-miR821a | AAGUCAUCAACAAAAAAGUUGAAU | 24 | Cleavage |
|  |  | osa-miR821b | AAGUCAUCAACAAAAAAGUUGAAU | 24 | Cleavage |
|  |  | osa-miR821c | AAGUCAUCAACAAAAAAGUUGAAU | 24 | Cleavage |
| *OsAPY8* | 11 | osa-miR5819 | AGGACGAGGGGAACGGCGGCG | 21 | Cleavage |
|  |  | osa-miR2927 | UGUCGUCGUCGAUGGAGCCCAUG | 23 | Cleavage |
|  |  | osa-miR5490 | UUGGAUUUUUAUUUAGGACGG | 21 | Translation |
|  |  | osa-miR1850.2 | UUGUGUGUGAACUAAACGUGG | 21 | Cleavage |
|  |  | osa-miR5809 | UCGUCGCCGGCGACCACAGC | 20 | Translation |
|  |  | osa-miR1424 | AUGCACACUGAUGCUGAUUGU | 21 | Cleavage |
|  |  | osa-miR1432-3p | CAGGUGUCAUCUCCCCUGAAC | 21 | Cleavage |
|  |  | osa-miR1858a | GAGAGGAGGACGGAGUGGGGC | 21 | Cleavage |
|  |  | osa-miR1858b | GAGAGGAGGACGGAGUGGGGC | 21 | Cleavage |
|  |  | osa-miR2099-3p | ACAAAGCUGUAGCGUUAUUC | 20 | Cleavage |
|  |  | osa-miR5789 | UGACUGAGCUUCGUUCGGUAU | 21 | Translation |
| *OsAPY9* | 29 | osa-miR160d-3p | GCGUGCGAGGAGCCAAGCAUG | 21 | Cleavage |
|  |  | osa-miR5075 | UUCUCCGUCGCCGCCGUCCGC | 21 | Cleavage |
|  |  | osa-miR5160 | CGAGAUCGAUGGUAUAUUUCUG | 22 | Translation |
|  |  | osa-miR5488 | UGAAGGCGACUGAUGAUUUCA | 21 | Cleavage |
|  |  | osa-miR1882a | AGAUUGCUUUCAAGGUCAUUUCUU | 24 | Cleavage |
|  |  | osa-miR1882b | AGAUUGCUUUCAAGGUCAUUUCUU | 24 | Cleavage |
|  |  | osa-miR1882c | AGAUUGCUUUCAAGGUCAUUUCUU | 24 | Cleavage |
|  |  | osa-miR1882d | AGAUUGCUUUCAAGGUCAUUUCUU | 24 | Cleavage |
|  |  | osa-miR1882e-5p | AGAUUGCUUUCAAGGUCAUUUCUU | 24 | Cleavage |
|  |  | osa-miR1882f | AGAUUGCUUUCAAGGUCAUUUCUU | 24 | Cleavage |
|  |  | osa-miR1882g | AGAUUGCUUUCAAGGUCAUUUCUU | 24 | Cleavage |
|  |  | osa-miR1882h | AGAUUGCUUUCAAGGUCAUUUCUU | 24 | Cleavage |
|  |  | osa-miR408-3p | CUGCACUGCCUCUUCCCUGGC | 21 | Translation |
|  |  | osa-miR5338 | UGAAGCUUCAGUUGGUUGUAU | 21 | Cleavage |
|  |  | osa-miR160a-3p | GCGUGCAAGGAGCCAAGCAUG | 21 | Cleavage |
|  |  | osa-miR160b-3p | GCGUGCAAGGAGCCAAGCAUG | 21 | Cleavage |
|  |  | osa-miR160e-3p | GCGUGCGAGGUGCCAAGCAUG | 21 | Translation |
|  |  | osa-miR166k-5p | GGUUUGUUGUCUGGCUCGAGG | 21 | Cleavage |
|  |  | osa-miR171a | UGAUUGAGCCGCGCCAAUAUC | 21 | Cleavage |
|  |  | osa-miR171i-3p | GGAUUGAGCCGCGUCAAUAUC | 21 | Cleavage |
|  |  | osa-miR2871a-3p | UAUUUUAGUUUCUAUGGUCAC | 21 | Cleavage |
|  |  | osa-miR2871b | UAUUUUAGUUUCUAUGGUCAC | 21 | Cleavage |
|  |  | osa-miR5148a | UGAGGGGUAGAAAUGUCAUAUCAU | 24 | Cleavage |
|  |  | osa-miR5148b | UGAGGGGUAGAAAUGUCAUAUCAU | 24 | Cleavage |
|  |  | osa-miR5148c | UGAGGGGUAGAAAUGUCAUAUCAU | 24 | Cleavage |
|  |  | osa-miR5543 | UAUGAAUGGUAUAUUUUCUUG | 21 | Cleavage |
|  |  | osa-miR5812 | AGCAACGAUUUUAAGAUUGUGGCA | 24 | Cleavage |
|  |  | osa-miR5819 | AGGACGAGGGGAACGGCGGCG | 21 | Cleavage |
|  |  | osa-miR5837.2 | GGUGAUGUGGAGCGUUCGGCA | 21 | Cleavage |
