# Supplementary material for: Identification, characterization of Apyrase (APY) gene family in rice (Oryza sativa) and analysis of the expression pattern under various stress conditions
Source: PLoS One. 2023 May 10;18(5):e0273592. doi: 10.1371/journal.pone.0273592 (PMC10171694; doi:10.1371/journal.pone.0273592)
Supplement: S5 Table — (DOCX) [file pone.0273592.s012.docx]

| **Gene Name** | **Alpha-Helix (%)** | **Beta-sheet (%)** | **Isolated Beta Bridge (%)** | **Turn (%)** | **Coil (%)** | **3-10 Helix (%)** | **Number of Predicted Membrane Spanning Motif (MSM)** | **Position of Motif(s) (bp)** |
| --- | --- | --- | --- | --- | --- | --- | --- | --- |
| OsAPY1 | 40.70 | 18.81 | 1.84 | 20.45 | 11.86 | 6.34 | 1 | 24 to 46 |
| OsAPY2 | 38.98 | 17.52 | 1.57 | 18.90 | 19.09 | 3.94 | 2 | 19 to 41, 467 to 484 |
| OsAPY3 | 38.33 | 19.70 | 2.14 | 24.63 | 11.13 | 4.07 | 1 | 21 to 43 |
| OsAPY4 | 40.22 | 20.85 | 0.74 | 15.13 | 18.82 | 4.24 | 2 | 45 to 62, 495 to 517 |
| OsAPY5 | 32.34 | 11.82 | 1.14 | 30.06 | 17.81 | 6.84 | 2 | 69 to 91, 548 to 570 |
| OsAPY6 | 38.69 | 18.80 | 0.73 | 21.53 | 16.61 | 3.65 | 2 | 10 to 32, 100 to 122 |
| OsAPY7 | 37.42 | 21.88 | 0.44 | 22.76 | 14.22 | 3.28 | 1 | 7 to 29 |
| OsAPY8 | 41.68 | 20.42 | 0.42 | 18.11 | 16.00 | 3.37 | 1 | 5 to 27 |
| OsAPY9 | 35.92 | 21.73 | 0.89 | 26.83 | 11.09 | 3.55 | 0 | N/A |
