# Supplementary material for: Identification, characterization of Apyrase (APY) gene family in rice (Oryza sativa) and analysis of the expression pattern under various stress conditions
Source: PLoS One. 2023 May 10;18(5):e0273592. doi: 10.1371/journal.pone.0273592 (PMC10171694; doi:10.1371/journal.pone.0273592)
Supplement: S6 Table — (DOCX) [file pone.0273592.s013.docx]

| **Protein Name** | **PROCHECK** | | | | **ERRAT** | **Z-Score** |
| --- | --- | --- | --- | --- | --- | --- |
|  | **Residues in most favoured region** | **Residues in the additional allowed region** | **Residues in generously allowed region** | **Residues in disallowed region** |  |  |
| OsAPY1 | 92.8 | 5.5 | 0.2 | 1.4 | 93.966 | -11.02 |
| OsAPY2 | 92.1 | 6.1 | 0.5 | 1.4 | 87.891 | -8.85 |
| OsAPY3 | 93.7 | 5 | 0.3 | 1 | 96.882 | -10.24 |
| OsAPY4 | 94.5 | 4.4 | 0.6 | 0.4 | 92.475 | -8.97 |
| OsAPY5 | 87.7 | 9.7 | 1.8 | 0.8 | 88.201 | -9.86 |
| OsAPY6 | 89 | 10 | 0 | 1 | 92.925 | -11.71 |
| OsAPY7 | 92.9 | 5.8 | 0 | 1.3 | 97.987 | -11.61 |
| OsAPY8 | 93.9 | 5.4 | 0 | 0.7 | 94.168 | -9.75 |
| OsAPY9 | 92.7 | 6 | 0.8 | 0.5 | 93.793 | -12.06 |
