# Supplementary material for: Identification, characterization of Apyrase (APY) gene family in rice (Oryza sativa) and analysis of the expression pattern under various stress conditions
Source: PLoS One. 2023 May 10;18(5):e0273592. doi: 10.1371/journal.pone.0273592 (PMC10171694; doi:10.1371/journal.pone.0273592)
Supplement: S7 Table — (DOCX) [file pone.0273592.s014.docx]

| **Docked Complex** | **Docking Score** |
| --- | --- |
| OsAPY1-ATP | -181.75 |
| OsAPY2-ATP | -199.05 |
| OsAPY3-ATP | -154.08 |
| OsAPY4-ATP | -152.53 |
| OsAPY5-ATP | -170.92 |
| OsAPY6-ATP | -170.22 |
| OsAPY7-ATP | -165.83 |
| OsAPY8-ATP | -160.91 |
| OsAPY9-ATP | -161.02 |
