# Supplementary material for: Identification, characterization of Apyrase (APY) gene family in rice (Oryza sativa) and analysis of the expression pattern under various stress conditions
Source: PLoS One. 2023 May 10;18(5):e0273592. doi: 10.1371/journal.pone.0273592 (PMC10171694; doi:10.1371/journal.pone.0273592)
Supplement: S8 Table — (DOCX) [file pone.0273592.s015.docx]

| **Protein Name** | **Hydrogen bond** | **Non-bond interaction** |
| --- | --- | --- |
| OsAPY1 | 10 | 118 |
| OsAPY2 | 3 | 134 |
| OsAPY3 | 11 | 120 |
| OsAPY4 | 7 | 119 |
| OsAPY5 | 6 | 159 |
| OsAPY6 | 6 | 111 |
| OsAPY7 | 4 | 111 |
| OsAPY8 | 8 | 98 |
| OsAPY9 | 11 | 166 |
