# Supplementary material for: Identification, characterization of Apyrase (APY) gene family in rice (Oryza sativa) and analysis of the expression pattern under various stress conditions
Source: PLoS One. 2023 May 10;18(5):e0273592. doi: 10.1371/journal.pone.0273592 (PMC10171694; doi:10.1371/journal.pone.0273592)
Supplement: S9 Table — (DOCX) [file pone.0273592.s016.docx]

| **OsAPY1** | | | | | | | |
| --- | --- | --- | --- | --- | --- | --- | --- |
| **Interacting Residue** | **Distance (Å)** | **Bond Type** | **Bond Category** | **Interacting Residue** | **Distance (Å)** | **Bond Type** | **Bond Category** |
| THR168 | 2.72 | Conventional Hydrogen Bond | Hydrogen Bond | ARG99 | 3.41 | Pi-Cation | Electrostatic |
| THR168 | 2.76 | Conventional Hydrogen Bond | Hydrogen Bond | SER381 | 2.03 | Unfavorable Bump | Unfavorable |
| GLN215 | 2.92 | Conventional Hydrogen Bond | Hydrogen Bond | TRP477 | 1.96 | Unfavorable Bump | Unfavorable |
| GLN245 | 2.26 | Conventional Hydrogen Bond | Hydrogen Bond | ASP92 | 4.57 | Unfavorable Negative-Negative | Unfavorable |
| SER285 | 2.24 | Conventional Hydrogen Bond | Hydrogen Bond | GLU211 | 2.51 | Unfavorable Negative-Negative | Unfavorable |
| ASP238 | 2.60 | Conventional Hydrogen Bond | Hydrogen Bond | GLU211 | 4.82 | Unfavorable Negative-Negative | Unfavorable |
| GLY97 | 2.48 | Conventional Hydrogen Bond | Hydrogen Bond | GLU211 | 3.16 | Unfavorable Negative-Negative | Unfavorable |
| ARG99 | 4.80 | Pi-Cation | Electrostatic | GLU211 | 5.01 | Unfavorable Negative-Negative | Unfavorable |
| **OsAPY2** | | | | | | | |
| **Interacting Residue** | **Distance (Å)** | **Bond Type** | **Bond Category** | **Interacting Residue** | **Distance (Å)** | **Bond Type** | **Bond Category** |
| ARG75 | 4.99 | Attractive Charge | Electrostatic | VAL436 | 2.10 | Carbon Hydrogen Bond | Hydrogen Bond |
| ARG75 | 2.69 | Conventional Hydrogen Bond | Hydrogen Bond | GLU207 | 2.64 | Carbon Hydrogen Bond | Hydrogen Bond |
| GLY209 | 2.89 | Conventional Hydrogen Bond | Hydrogen Bond | THR350 | 3.00 | Carbon Hydrogen Bond | Hydrogen Bond |
| GLN214 | 2.52 | Conventional Hydrogen Bond | Hydrogen Bond | ASN352 | 2.48 | Pi-Donor Hydrogen Bond | Hydrogen Bond |
| PHE355 | 2.94 | Conventional Hydrogen Bond | Hydrogen Bond | GLU351 | 2.54 | Pi-Sigma | Hydrophobic |
| TRP438 | 2.71 | Conventional Hydrogen Bond | Hydrogen Bond | GLU351 | 1.84 | Unfavorable Bump | Unfavorable |
| VAL436 | 2.50 | Conventional Hydrogen Bond | Hydrogen Bond | GLU351 | 2.09 | Unfavorable Bump | Unfavorable |
| GLY209 | 2.89 | Carbon Hydrogen Bond | Hydrogen Bond | GLU351 | 4.04 | Unfavorable Negative-Negative | Unfavorable |
| TRP438 | 2.22 | Carbon Hydrogen Bond | Hydrogen Bond | GLU207 | 2.59 | Unfavorable Acceptor-Acceptor | Unfavorable |
| ASN429 | 2.88 | Carbon Hydrogen Bond | Hydrogen Bond | THR350 | 2.93 | Unfavorable Acceptor-Acceptor | Unfavorable |
| **OsAPY3** | | | | | | | |
| **Interacting Residue** | **Distance (Å)** | **Bond Type** | **Bond Category** | **Interacting Residue** | **Distance (Å)** | **Bond Type** | **Bond Category** |
| ARG153 | 4.90 | Attractive Charge | Electrostatic | GLU111 | 2.91 | Conventional Hydrogen Bond | Hydrogen Bond |
| SER110 | 3.16 | Conventional Hydrogen Bond | Hydrogen Bond | SER76 | 3.70 | Carbon Hydrogen Bond | Hydrogen Bond |
| THR149 | 3.26 | Conventional Hydrogen Bond | Hydrogen Bond | THR149 | 3.34 | Carbon Hydrogen Bond | Hydrogen Bond |
| GLY222 | 2.81 | Conventional Hydrogen Bond | Hydrogen Bond | GLY151 | 3.37 | Carbon Hydrogen Bond | Hydrogen Bond |
| GLY223 | 2.39 | Conventional Hydrogen Bond | Hydrogen Bond | LEU273 | 4.40 | Pi-Alkyl | Hydrophobic |
| GLY223 | 2.39 | Conventional Hydrogen Bond | Hydrogen Bond | LEU273 | 5.42 | Pi-Alkyl | Hydrophobic |
| SER224 | 2.65 | Conventional Hydrogen Bond | Hydrogen Bond | LYS301 | 4.60 | Pi-Alkyl | Hydrophobic |
| SER224 | 3.24 | Conventional Hydrogen Bond | Hydrogen Bond | SER110 | 2.06 | Unfavorable Bump | Unfavorable |
| GLY303 | 3.23 | Conventional Hydrogen Bond | Hydrogen Bond | TYR302 | 2.16 | Unfavorable Bump | Unfavorable |
| **OsAPY4** | | | | | | | |
| **Interacting Residue** | **Distance (Å)** | **Bond Type** | **Bond Category** | **Interacting Residue** | **Distance (Å)** | **Bond Type** | **Bond Category** |
| TYR73 | 2.54 | Conventional Hydrogen Bond | Hydrogen Bond | GLU151 | 1.80 | Unfavorable Bump | Unfavorable |
| THR152 | 2.88 | Conventional Hydrogen Bond | Hydrogen Bond | GLU72 | 4.31 | Unfavorable Negative-Negative | Unfavorable |
| ARG155 | 3.20 | Conventional Hydrogen Bond | Hydrogen Bond | GLU72 | 5.25 | Unfavorable Negative-Negative | Unfavorable |
| GLN189 | 2.80 | Conventional Hydrogen Bond | Hydrogen Bond | GLU72 | 5.44 | Unfavorable Negative-Negative | Unfavorable |
| GLU72 | 3.57 | Carbon Hydrogen Bond | Hydrogen Bond | GLU151 | 4.41 | Unfavorable Negative-Negative | Unfavorable |
| SER148 | 3.22 | Carbon Hydrogen Bond | Hydrogen Bond | GLU151 | 5.03 | Unfavorable Negative-Negative | Unfavorable |
| GLN187 | 3.38 | Pi-Donor Hydrogen Bond | Hydrogen Bond | GLU151 | 3.29 | Unfavorable Negative-Negative | Unfavorable |
| TYR483 | 4.05 | Pi-Donor Hydrogen Bond | Hydrogen Bond | ASP153 | 5.34 | Unfavorable Negative-Negative | Unfavorable |
| GLU72 | 2.11 | Unfavorable Bump | Unfavorable | TRP192 | 2.78 | Unfavorable Donor-Donor | Unfavorable |
| **OsAPY5** | | | | | | | |
| **Interacting Residue** | **Distance (Å)** | **Bond Type** | **Bond Category** | **Interacting Residue** | **Distance (Å)** | **Bond Type** | **Bond Category** |
| TRP247 | 1.56 | Conventional Hydrogen Bond | Hydrogen Bond | SER261 | 2.43 | Carbon Hydrogen Bond | Hydrogen Bond |
| THR265 | 2.56 | Conventional Hydrogen Bond | Hydrogen Bond | GLU281 | 2.16 | Carbon Hydrogen Bond | Hydrogen Bond |
| GLY267 | 2.37 | Conventional Hydrogen Bond | Hydrogen Bond | ASP283 | 1.84 | Carbon Hydrogen Bond | Hydrogen Bond |
| GLU281 | 2.86 | Conventional Hydrogen Bond | Hydrogen Bond | GLU281 | 4.54 | Pi-Anion | Electrostatic |
| SER300 | 2.65 | Conventional Hydrogen Bond | Hydrogen Bond | PHE432 | 3.79 | Pi-Anion | Electrostatic |
| GLN302 | 2.09 | Conventional Hydrogen Bond | Hydrogen Bond | GLU281 | 2.96 | Pi-Lone Pair | Other |
| SER261 | 2.11 | Carbon Hydrogen Bond | Hydrogen Bond | GLU281 | 1.73 | Unfavorable Bump | Unfavorable |
| GLY267 | 2.73 | Carbon Hydrogen Bond | Hydrogen Bond | GLU281 | 2.09 | Unfavorable Bump | Unfavorable |
| GLY267 | 2.76 | Carbon Hydrogen Bond | Hydrogen Bond | HIS429 | 2.21 | Unfavorable Bump | Unfavorable |
| PHE280 | 2.68 | Carbon Hydrogen Bond | Hydrogen Bond | HIS429 | 1.63 | Unfavorable Bump | Unfavorable |
| THR282 | 2.40 | Carbon Hydrogen Bond | Hydrogen Bond | SER261 | 1.42 | Unfavorable Bump | Unfavorable |
| HIS429 | 3.00 | Carbon Hydrogen Bond | Hydrogen Bond | ASP283 | 5.23 | Unfavorable Negative-Negative | Unfavorable |
| GLY430 | 2.73 | Carbon Hydrogen Bond | Hydrogen Bond | SER300 | 2.50 | Unfavorable Acceptor-Acceptor | Unfavorable |
| **OsAPY6** | | | | | | | |
| **Interacting Residue** | **Distance (Å)** | **Bond Type** | **Bond Category** | **Interacting Residue** | **Distance (Å)** | **Bond Type** | **Bond Category** |
| ARG476 | 4.60 | Attractive Charge | Electrostatic | GLU470 | 4.59 | Pi-Anion | Electrostatic |
| ARG476 | 4.04 | Attractive Charge | Electrostatic | ASN194 | 4.07 | Pi-Donor Hydrogen Bond | Hydrogen Bond |
| ARG476 | 2.98 | Attractive Charge | Electrostatic | LYS154 | 5.09 | Pi-Alkyl | Hydrophobic |
| ARG476 | 3.09 | Attractive Charge | Electrostatic | TYR204 | 1.74 | Unfavorable Bump | Unfavorable |
| GLN200 | 2.89 | Conventional Hydrogen Bond | Hydrogen Bond | TYR263 | 1.79 | Unfavorable Bump | Unfavorable |
| THR473 | 3.31 | Conventional Hydrogen Bond | Hydrogen Bond | GLU470 | 2.06 | Unfavorable Bump | Unfavorable |
| ARG476 | 2.87 | Conventional Hydrogen Bond | Hydrogen Bond | THR473 | 1.78 | Unfavorable Bump | Unfavorable |
| ARG476 | 2.90 | Conventional Hydrogen Bond | Hydrogen Bond | ARG476 | 1.78 | Unfavorable Bump | Unfavorable |
| GLY474 | 3.58 | Carbon Hydrogen Bond | Hydrogen Bond | ARG476 | 0.74 | Unfavorable Bump | Unfavorable |
| GLU470 | 2.52 | Carbon Hydrogen Bond | Hydrogen Bond | GLU262 | 2.70 | Unfavorable Acceptor-Acceptor | Unfavorable |
| LYS154 | 4.70 | Pi-Cation | Electrostatic | GLU470 | 2.65 | Unfavorable Acceptor-Acceptor | Unfavorable |
| LYS154 | 3.96 | Pi-Cation;Pi-Donor Hydrogen Bond | Hydrogen Bond;Electrostatic | GLU470 | 2.32 | Unfavorable Acceptor-Acceptor | Unfavorable |
| GLU470 | 4.21 | Pi-Anion | Electrostatic |  |  |  |  |
| **OsAPY7** | | | | | | | |
| **Interacting Residue** | **Distance (Å)** | **Bond Type** | **Bond Category** | **Interacting Residue** | **Distance (Å)** | **Bond Type** | **Bond Category** |
| GLN176 | 2.50 | Conventional Hydrogen Bond | Hydrogen Bond | LYS452 | 3.90 | Pi-Cation;Pi-Donor Hydrogen Bond | Hydrogen Bond;Electrostatic |
| TYR180 | 2.56 | Conventional Hydrogen Bond | Hydrogen Bond | GLU446 | 3.05 | Pi-Anion | Electrostatic |
| GLU456 | 2.48 | Conventional Hydrogen Bond | Hydrogen Bond | LYS452 | 5.49 | Pi-Alkyl | Hydrophobic |
| GLU238 | 2.63 | Conventional Hydrogen Bond | Hydrogen Bond | VAL171 | 1.95 | Unfavorable Bump;Unfavorable Acceptor-Acceptor | Unfavorable |
| GLU446 | 3.50 | Pi-Anion | Electrostatic |  |  |  |  |
| **OsAPY8** | | | | | | | |
| **Interacting Residue** | **Distance (Å)** | **Bond Type** | **Bond Category** | **Interacting Residue** | **Distance (Å)** | **Bond Type** | **Bond Category** |
| ARG279 | 5.23 | Attractive Charge | Electrostatic | ALA38 | 2.34 | Conventional Hydrogen Bond | Hydrogen Bond |
| SER42 | 2.38 | Conventional Hydrogen Bond | Hydrogen Bond | ALA38 | 3.35 | Carbon Hydrogen Bond | Hydrogen Bond |
| SER79 | 3.31 | Conventional Hydrogen Bond | Hydrogen Bond | ASP413 | 4.97 | Pi-Anion | Electrostatic |
| ARG279 | 3.21 | Conventional Hydrogen Bond | Hydrogen Bond | SER42 | 2.03 | Unfavorable Bump | Unfavorable |
| TYR302 | 3.11 | Conventional Hydrogen Bond | Hydrogen Bond | GLN276 | 1.99 | Unfavorable Bump | Unfavorable |
| ALA38 | 3.27 | Conventional Hydrogen Bond | Hydrogen Bond | ARG279 | 2.42 | Unfavorable Donor-Donor | Unfavorable |
| **OsAPY9** | | | | | | | |
| **Interacting Residue** | **Distance (Å)** | **Bond Type** | **Bond Category** | **Interacting Residue** | **Distance (Å)** | **Bond Type** | **Bond Category** |
| ARG130 | 3.26 | Salt Bridge | Hydrogen Bond;Electrostatic | ARG57 | 4.56 | Pi-Cation | Electrostatic |
| ARG130 | 3.41 | Attractive Charge | Electrostatic | ASP50 | 2.77 | Pi-Anion | Electrostatic |
| ARG130 | 5.19 | Attractive Charge | Electrostatic | ARG57 | 4.50 | Pi-Alkyl | Hydrophobic |
| SER53 | 2.79 | Conventional Hydrogen Bond | Hydrogen Bond | THR126 | 2.04 | Unfavorable Bump;Conventional Hydrogen Bond | Unfavorable |
| ALA127 | 3.04 | Conventional Hydrogen Bond | Hydrogen Bond | ALA127 | 1.86 | Unfavorable Bump | Unfavorable |
| GLY128 | 3.32 | Conventional Hydrogen Bond | Hydrogen Bond | ALA127 | 2.21 | Unfavorable Bump | Unfavorable |
| GLY200 | 3.18 | Conventional Hydrogen Bond | Hydrogen Bond | SER201 | 2.24 | Unfavorable Bump | Unfavorable |
| SER201 | 2.54 | Conventional Hydrogen Bond | Hydrogen Bond | SER201 | 2.10 | Unfavorable Bump;Conventional Hydrogen Bond | Unfavorable |
| ASP50 | 2.61 | Conventional Hydrogen Bond | Hydrogen Bond | SER201 | 1.25 | Unfavorable Bump | Unfavorable |
| GLY55 | 2.23 | Conventional Hydrogen Bond | Hydrogen Bond | GLY52 | 3.28 | Unfavorable Donor-Donor | Unfavorable |
| ARG57 | 4.17 | Pi-Cation | Electrostatic | GLU169 | 2.50 | Unfavorable Acceptor-Acceptor | Unfavorable |
