# Supplementary material for: Identification, characterization of Apyrase (APY) gene family in rice (Oryza sativa) and analysis of the expression pattern under various stress conditions
Source: PLoS One. 2023 May 10;18(5):e0273592. doi: 10.1371/journal.pone.0273592 (PMC10171694; doi:10.1371/journal.pone.0273592)
Supplement: S10 Table — (DOCX) [file pone.0273592.s017.docx]

| **Gene Name** | **Forward primer** | **Reverse primer** |
| --- | --- | --- |
| *OsAPY1* | GGTTCTGTCCAAATGGCTTA | TCCAACATACAGTTGCGGTA |
| *OsAPY2* | CAAACTATGCTCTTGGCACA | AAGGATTCTTGTGCAGCATT |
| *OsAPY3* | ATCTTGGAGGTGGATCTGTG | AGCTGTAACCTTTGCCATTG |
| *OsAPY4* | TGCTTTGCTGCATGATACTC | AAATAGAACGACAGCGGAAA |
| *OsAPY5* | ACACACGCCTGCTTTTCTAT | GATGAGGTGCCAAGCATATT |
| *OsAPY6* | CTCTGCTTGATAAGGCCATC | TCCTTCTTGAGATCCCGTAA |
| *OsAPY7* | GTCCACACCAAGACCAAGTT | AGCACTTCTGGAGCATTAGC |
| *OsAPY8* | TCAGAAAAGAACTCCCCTCA | CCTCCTAACTTCCCCAAAAG |
| *OsAPY9* | ACGTCTTCAGGTTCGACAAC | TAAGTCTGAGTCCGGCAGTT |
